# How do consumers perceive virtual agents with behavioral realism? The role of loneliness and trust

**DOI:** 10.3389/fpsyg.2026.1676234

**Published:** 2026-02-26

**Authors:** Binbin Sun, Yutong Chen

**Affiliations:** School of Business, Qingdao University, Qingdao, China

**Keywords:** behavioral realism, response type, state loneliness, trait loneliness, virtual agent

## Abstract

**Introduction:**

Virtual agents with realism have been widely promoted as a potential solution to the pervasive global problem of loneliness by providing companionship. However, prior research and practical applications have produced inconsistent and sometimes contradictory findings. To elucidate the mechanisms underlying this discrepancy, this study further explores how virtual assistants with behavioral realism influence the usage willingness of lonely consumers and the underlying mechanisms of this influence.

**Methods:**

Using a scenario-based experimental approach, this research conducted three single-factor between-subjects experiments manipulating response type (scripted vs. natural). Data were analyzed using SPSS 25.0 and PROCESS 4.1. Study 1 examined the main effect of response type on willingness to use. Study 2 tested the mediating roles of cognitive trust and affective trust. Study 3 investigated the moderating role of loneliness type in the relationship between response type and willingness to use, as well as the conditional mediation pathways through which this moderation operates.

**Results:**

The results reveal distinct preference patterns across different types of loneliness. Consumers with high state loneliness show greater willingness to use virtual agents with natural responses due to enhanced social connection and affective trust. In contrast, consumers with high trait loneliness tend to prefer virtual agents with scripted responses, as natural responses often elicit social avoidance and reduce affective trust.

**Discussion:**

By jointly examining the types of loneliness and virtual agent characteristics, this research helps reconcile previously inconsistent findings in the human–AI interaction literature. The results offer important implications for the design and deployment of virtual agents, suggesting that tailoring behavioral realism to users' loneliness profiles can enhance willingness and effectiveness among different types of lonely consumers.

## 1 Introduction

According to a survey conducted by Gallup and Meta, over one billion people worldwide are struggling with loneliness (Maese, 2023). The World Health Organization declared loneliness to be an “urgent global health threat” on November 15, 2023. The widespread prevalence of loneliness has drawn the attention of scholars, with some suggesting that humanoid virtual agents capable of dialogue could serve consumers while providing emotional value to those experiencing loneliness, offering companionship and alleviating feelings of isolation (Hu et al., 2023; Razavi et al., 2022). Other studies have noted that consumers with strong feelings of loneliness tend to hold negative attitudes toward social behavior, and humanoid virtual agents may evoke avoidance of interpersonal social interaction, leading them to be unwilling to use such agents (Dang and Liu, 2023; Yu and Fan, 2024). In this study, a virtual agent refers to an AI-controlled digital entity with a human-like appearance that is capable of interacting in real time.

With the advancement of artificial intelligence technology, virtual agents are rapidly becoming widespread as intelligent customer service representatives across various industries (Yao et al., 2023). These virtual assistants, which simulate human conversation, are expected to reach a market size of over US$100 billion by 2032 (Research Nester, 2024). Currently, highly anthropomorphic virtual agents (such as Air New Zealand's “Sophie”) can already provide an interaction experience close to that of real humans (Miao et al., 2022). However, the actual effectiveness of virtual agents remains controversial. For example, Kiehl's launched a digital skincare expert named EVE, whose appearance and behavior are highly human-like and which has been well-received by consumers (UneeQ, 2022). However, IKEA had to discontinue its virtual agent Anna due to users' overly high expectations of its capabilities, which led to conversations exceeding the preset scripts in the programming (Brandtzaeg and Følstad, 2018). It is evident that due to deficiencies in behavioral realism, consumers may pause or refuse to continue interacting with chatbots. Therefore, companies need to identify the characteristics of virtual agents related to consumer interactions that enhance behavioral realism, thereby better stimulating consumer willingness to use. This influence can be explained through the “Computers Are Social Actors” (CASA) paradigm. This paradigm assumes that when computer interfaces exhibit social characteristics, humans interact with them in ways that resemble human–human interaction, representing a natural and instinctive response (Nass et al., 1994). Notably, according to the Computers Are Social Actors paradigm, the relationship between virtual agent characteristics and consumer willingness to use is also influenced by user attributes (Krämer et al., 2015). This study focuses on loneliness as a user attribute.

These mixed findings suggest that a deeper exploration of the theoretical logic and mechanisms underlying the willingness to use virtual agents, particularly among consumers experiencing loneliness, is necessary. This paper cuts across the study of loneliness to provide a theory that explains why and by whom humanoid virtual agents are popular. Our study relates to research gaps in the following three areas.

First, this paper is related to the study of response types of virtual agents. Response types are an important interaction feature of virtual agents, as they may shape users' perceptions of how naturally an agent behaves and, in turn, influence consumers' willingness to engage in conversation with it. From the current research, it seems that scripted responses cause consumers to perceive virtual agents as lacking emotion and having limited understanding and use of language (Jiang et al., 2022). In contrast, advances in AI technology have enabled virtual agents to generate more natural responses, which may enhance users' perceptions of behavioral realism during interactions (Miao et al., 2022; Yang et al., 2024; Yu et al., 2024). However, response types have received limited direct attention as a focal research topic, and our study aims to bridge this gap.

Second, prior consumer behavior studies have often treated loneliness as a singular variable. In contrast, this study categorizes loneliness into state and trait loneliness based on psychological research (Jones et al., 1990). State loneliness refers to a consumer's short-term isolation due to specific situational factors (e.g., social exclusion or isolation measures). In contrast, trait loneliness is a stable personality attribute (van Roekel et al., 2018). Consumers experiencing state loneliness tend to increase their social connections with others to mitigate its negative effects, while those with trait loneliness may avoid social interactions altogether (Cacioppo and Cacioppo, 2018). Therefore, the type of loneliness can distinctly influence consumers' social intentions, yet it remains unclear how these varying impacts might further affect their willingness to utilize virtual agents.

Third, our research examines trust in human-AI interactions. Prior studies have confirmed that trust can emerge in human-AI interactions and that the form realism of intelligent machines can enhance this trust (Chen et al., 2024; Fatima et al., 2024). However, there is limited evidence on how behavioral realism influences trust. Additionally, psychological research indicates that while loneliness can diminish consumers' expectations of trustworthiness in others, lonelier consumers may also exhibit increased trust behaviors (Bellucci and Park, 2024). Thus, the effects of different types of loneliness on trust in interactions with virtual agents that exhibit varying levels of behavioral realism remain underexplored.

Our research findings contribute significantly to both theoretical and practical domains. Theoretically, first, this study focuses on response type as a key factor to further clarify the relationship between the behavioral realism of virtual agents and consumers' willingness to use them, and videos will be used as the experimental materials to increase the authenticity. Secondly, this study explores the different effects of state loneliness and trait loneliness on willingness to use in virtual agent service situations based on prior categorizations of loneliness, providing further explanations for the divergent results on loneliness in prior human-AI interaction studies. Finally, this study extends research on trust in human–AI interactions by identifying a novel link between loneliness and trust formation. From a practical perspective, our research helps businesses and developers understand the significance of consumers' types of loneliness in virtual agent service scenarios. When employing virtual agents for services, it is essential to differentiate users based on their types of loneliness and effectively leverage various response types to enhance user engagement.

The rest of our study is arranged as follows. We review the past related literature in Section 2 and outline the hypotheses in Section 3. Then, this study presents three empirical studies and analyzes the findings in Section 4: Study 1 investigates how scripted vs. natural responses influence consumers' willingness to use; Study 2 explores the mediating roles of cognitive trust and affective trust between response types and willingness to use; Study 3 explores the moderating effects of state and trait loneliness on willingness to use and trust. Finally, Section 5 of this study concludes our findings' theoretical implications and practical implications, identifies the limitations of the study, and outlines directions for future research.

## 2 Literature review

### 2.1 Behavioral realism of virtual agents

In this study, virtual agents can be defined as AI-controlled virtual digital representatives with anthropomorphic appearances and real-time interactive capabilities (Miao et al., 2022). The design of virtual agents can be categorized into two major elements. The first element is form realism, which refers to the degree of similarity between the virtual agent and human appearance (Nowak and Fox, 2018). Many prior studies have explored the relationship between the form realism of virtual agents and consumers' willingness to use them (Qiu and Benbasat, 2005; Xie et al., 2024; Yang et al., 2024; Yao et al., 2023). The second type of element is behavioral realism, which reflects how virtual agents replicate human behaviors in the real world. Key design elements include communication methods, response types, and social presence (Miao et al., 2022). Among these elements, the present study focuses specifically on response types. The response types of virtual agents are subdivided into scripted and natural. Scripted response refers to virtual agents that primarily provide replies from a predetermined set of stored responses. In contrast, virtual agents with natural responses can engage in “relatively free-flowing conversations, utilizing acceptable vocabulary and grammar, can be contextualized and responded to appropriately” (Burden and Savin-Baden, 2019; Miao et al., 2022). According to Kim et al. (2024), scripted and natural responses are often associated with different levels of perceived behavioral realism in prior research.

Prior research suggests that certain behavioral design elements of virtual agents are associated with more favorable user responses. For example, van Pinxteren et al. (2023) discovered that virtual agents utilizing social-oriented communication produced higher levels of perceived rapport than those using task-oriented communication, resulting in greater satisfaction and willingness to reuse the services. Other studies have similarly highlighted that the behaviors of virtual agents, such as self-disclosure, reciprocal behavior (Lee and Choi, 2017), social cues (Yan et al., 2024), emotional expression (Xie et al., 2024; Yu et al., 2024), and product usage behavior (Yang et al., 2024), significantly influence purchasing and willingness to use. Although these studies focus on diverse behavioral features, they collectively suggest that how virtual agents behave during interaction can influence user outcomes. In addition, Kim et al. (2024) studied the moderating effect of response type on the influence of form realism on consumer willingness to follow in the context of virtual influencers. Scripted responses were described as relatively standardized and brief, such as “thank you” and “kiss”; natural responses, on the other hand, involved more personalized and emotionally expressive interaction styles, such as replying to a fan's comment about a vote with “sooo happy y'all voted for that one and didn't make me show up not ready to STUNT. Bernice was a little mad though.” Although response types have been studied in the field of virtual influencers, there is currently a lack of research on response types in the field of virtual agents, even though response types are crucial in the interaction between consumers and virtual agents. Our study will fill this gap by focusing on the impact of different response types on the willingness of consumers to use virtual agents. In addition to this, this study will draw on the research of Kim D. Y. et al. (2023) and use video as experimental material to increase the immersion and make up for the lack of prior studies that used pictures or textual experimental material in terms of creating realistic scenarios. Research indicates that videos as experimental stimuli can create a more immersive experimental environment, eliciting participant psychological responses similar to those observed in real-service-scenario studies (Bateson and Hui, 1992).

Prior studies on virtual agents have mostly focused on scripted responses, with a few studies using real people to simulate virtual agents that exhibit more natural and flexible response behaviors as experimental stimuli. Virtual agents with scripted responses can only select replies from a pre-set corpus (van Pinxteren et al., 2023) and even provide limited dialogue options (Pot et al., 2017). When they fail to understand user queries or lack suitable responses, they may be unable to offer appropriate replies, leading to negative experiences. For example, Schuetzler et al. (2018) found that scripted virtual interviewers tend to elicit more negative reactions from consumers. However, advancements in AI are poised to change this situation fundamentally (Kim J. et al., 2023; Khennouche et al., 2024), enabling virtual agents to generate more flexible and contextually appropriate responses. Virtual agents powered by large language models can be trained on dialogue datasets to communicate with customers (Bansal et al., 2024), thereby enabling a different style of response generation rather than merely expanding functional capabilities. As a result, such agents can produce responses that are often perceived as more flexible and contextually appropriate, compared with predefined scripted responses (Khennouche et al., 2024). Despite these valuable insights, existing research has yet to conduct comparative studies between scripted and natural virtual agents' responses. Given the technological advancements that have led to the emergence of more naturally responding virtual agents, it is essential to realign the research focus accordingly.

### 2.2 Cognitive trust and affective trust

As AI agents continue to evolve, many tasks that were previously performed by humans are being taken over by AI, leading to an increase in the perceived risk in the human-AI relationship (Kozyreva et al., 2021; Park and Jones-Jang, 2023). Trust can help consumers overcome the insecurity associated with these risks, allowing them to use new technologies with confidence and, in some cases, develop dependence on them (Gillath et al., 2021). In recent years, research on trust in human–AI interactions has been extended to education (Zhang et al., 2024), e-government (Wang Y.-F. et al., 2024), and online shopping (Chakraborty et al., 2024). Furthermore, trust has been empirically validated as a critical predictor of the adoption of virtual agents (Bach et al., 2024; Shahzad et al., 2024) and has facilitated user adoption and use of virtual agents in several domains (Ho and Cheung, 2024; Liu et al., 2025; Park et al., 2024; Yan et al., 2024). Conversely, when consumers distrust virtual agents, their loyalty toward these agents is likely to be compromised (Rajaobelina et al., 2021).

McAllister (1995) conceptualizes trust as categorized into cognitive trust and affective trust. For this study, cognitive trust refers to the trustor's rational evaluation of the trustee's ability to fulfill the obligations understood by the trustor (McAllister, 1995). This form of trust primarily stems from users' perceptions of the virtual agent, including three dimensions: ability, benevolence, and integrity, which influence whether users can develop cognitive trust in virtual agents (Wang et al., 2016). In contrast, affective trust refers to the emotional bond between the trustor and the trustee due to the perceived closeness and warmth of psychological attachment (Gkinko and Elbanna, 2023). Although traditionally, affective trust is thought to require time to form (McAllister, 1995), recent research suggests that its emotional basis can sometimes form rapidly (Legood et al., 2023). In the field of intelligent customer service, the impact of virtual agent attributes on cognitive and affective trust primarily revolves around the presence or absence of explanatory features, virtual agent interface design, immediacy, and transparency (Suen and Hung, 2023; Wang et al., 2016). Our research will further explore the effects of virtual agents' response types on cognitive and affective trust from the perspective of behavioral realism.

### 2.3 State loneliness and trait loneliness

Loneliness is “a subjective feeling resulting from a discrepancy between expectations and actual social relationships in terms of companionship, connection, or intimacy” (Buecker et al., 2020). However, existing research has not reached a consensus regarding how loneliness influences individuals' tendencies to seek social connections. On the one hand, individuals experiencing loneliness are prone to perceive their existing social relationships as insufficient, which may lead to a strong willingness to seek social connections and companionship (Bellucci, 2020). With advancements in AI, highly lonely people have found social and emotional outlets in virtual networks (Zhao and Kou, 2024). Some studies suggest that AI agents such as Alexa may serve as safe social options, helping lonely consumers manage social difficulties and thereby alleviating feelings of loneliness (Hu et al., 2023; Lai et al., 2025). Additionally, virtual agents have been employed to alleviate depression and loneliness among elderly consumers in hospitals and long-term care facilities, helping to reduce social anxiety through conversation (Bott et al., 2019; Fraune et al., 2022; Razavi et al., 2022).

On the other hand, some studies have shown that due to past socialization failures in real life, individuals with higher levels of loneliness tend to enter a state of self-protection during socialization (Cacioppo and Cacioppo, 2018). This tendency may deepen negative perceptions of social connections and maintain greater social distance (Saporta et al., 2021), resulting in more uncooperative and adversarial responses during interpersonal communication (Anderson and Martin, 1995). In such situations, lonely consumers may prefer to passively endure their loneliness rather than proactively seek to improve their social relationships (Cacioppo and Patrick, 2008). Under such conditions, the degree of loneliness has an impact on attitudes toward companion robots, with those with higher levels of loneliness being less inclined to use them (Dang and Liu, 2023; Yu and Fan, 2024). In summary, prior research reveals conflicting views regarding how loneliness influences consumer willingness to engage with virtual agents.

Different types of loneliness may influence consumers' willingness to engage with virtual agents in different ways. According to the Evolutionary Theory of Loneliness, to cope with and adapt to loneliness, a consumer's biological early warning system evokes dual psychological motives at the same time, convergent motives to restore self-differentiation and avoidance motives for self-protection (Cacioppo and Cacioppo, 2018). Whether one is in a state of loneliness for a long time is the key factor that determines which psychological motive dominates (Saporta et al., 2021). Therefore, for consumers with state loneliness, as their experience of loneliness is relatively short-lived, the convergent motive to restore self-differentiation is dominant, which may generate psychological needs such as social connection, and the tendency to seek interpersonal interaction to adapt to the negative effects of loneliness as soon as possible (Lai et al., 2025). In contrast, for consumers with trait loneliness, prolonged exposure to loneliness leads to the dominance of avoidance motives for self-protection, which generates implicit vigilance, increases risk perception of the external environment, and generates social avoidance needs (Cacioppo and Cacioppo, 2018). Thus, different types of loneliness may give rise to consumers' social intentions. However, it remains to be verified whether this classification of loneliness continues to impact consumers' willingness to engage with virtual agents. Accordingly, this study examines how state loneliness and trait loneliness differentially influence consumers' willingness to use virtual agents.

Additionally, prior research has generally suggested that loneliness can lead to diminished expectations of others' trust (Sease et al., 2024; Yuan et al., 2022). However, Bellucci and Park (2024) recently noted that lonelier consumers are more likely to trust others. Our study will also explore the relationship between different types of loneliness and trust within interactions with virtual agents of varying response types.

## 3 Hypotheses

### 3.1 Response type of virtual agent and consumers' willingness to use

According to the CASA paradigm, the social influence in human-AI interactions is like that in human-to-human interactions (Reeves and Nass, 1996). The paradigm is that people view computer-generated digital entities as social others and apply real-world experiences to the digital world, making social judgments and attributions from the characteristics of their virtual agents as they would with a real person (Nass et al., 1994; Nass and Moon, 2000). In other words, when virtual agents display characteristics that users perceive as more human-like during interaction, consumers are more likely to regard these agents as social others and interact with them according to the rules of interpersonal communication (Ham et al., 2024). This leads to better behavioral intentions and greater trust (Munnukka et al., 2022; Crolic et al., 2022; Jan et al., 2023). Moreover, this realism is not limited to the appearance of virtual agents but extends to behaviors (Zhang and Wang, 2023; Dabiran et al., 2024), including language cues, empathy, and emotional expression (Adam et al., 2021; Kim T. et al., 2023; Guo et al., 2025).

Through our review of the literature, we found that virtual agents with scripted responses often exhibit lower interactivity and relevance in communication, frequently providing rigid replies or limited options, which may lead users to perceive these agents as having lower behavioral realism (Pot et al., 2017; van Pinxteren et al., 2023). In contrast, virtual agents with natural responses tend to demonstrate higher interactivity and contextual relevance in communication, enabling them to address a wider range of user inquiries. As a result, such agents can generate responses that are often perceived as more flexible and contextually appropriate compared with predefined scripted responses (Bansal et al., 2024; Khennouche et al., 2024).

Building on the CASA paradigm and existing literature, in the context of customer service, we propose that consumers will perceive a more remarkable similarity with natural virtual agents than scripted ones, enhancing their willingness to use them as intelligent customer service representatives. Hence, we present the following hypothesis:

*H1*: Consumers have more willingness to use virtual agents with natural responses than virtual agents with scripted responses.

### 3.2 The mediating role of cognitive trust and affective trust

According to the CASA paradigm, interactions between consumers and computers and other new media technologies inherently tend to be social (Nass et al., 1995). Humans attribute social characteristics (i.e., trustworthiness and attractiveness) to AI technology during interactions (Edwards et al., 2019), and this attribution is related to the characteristics of technologies such as computers (Nass and Moon, 2000). Prior studies have demonstrated that users are more willing to connect with and trust virtual agents that are perceived as exhibiting higher behavioral realism (Fatima et al., 2024; Munnukka et al., 2022; Chang and Park, 2024), which may arise from cues such as emotionally expressive communication, casual conversational styles, and natural behavioral displays (Wei et al., 2025). In other words, differences in response type may influence the extent to which users develop trust in virtual agents. On the one hand, more natural conversational responses may signal greater interactive capability, thereby potentially enhancing users' cognitive trust (Ham et al., 2024; Vanman and Kappas, 2019). On the other hand, emotionally expressive elements embedded in natural responses may facilitate emotional connections, thereby promoting affective trust (Ham et al., 2024).

Based on extensive empirical evidence, trust has been identified as a key predictor of technology adoption (Bach et al., 2024). Trust in human-AI interactions has been shown to have a direct impact on consumers' intentions to interact with AI (Chi et al., 2023). Consistent with this view, numerous studies indicate that users are more likely to adopt virtual agents when a sufficient level of trust has been established (Chang and Park, 2024; Choung et al., 2023; Ho and Cheung, 2024; Kim et al., 2021; Le et al., 2023). Conversely, a lack of trust in virtual agents can hinder users' adoption and continued use of such systems. Based on this, we propose the following hypothesis:

*H2*: Consumers' cognitive trust and affective trust mediate the influence of response types on their willingness to use virtual agents. Specifically, for virtual agents with natural responses (vs. scripted responses), consumers' cognitive trust and affective trust increase, and thus have a stronger willingness to use the virtual agent.

### 3.3 The moderating effects of loneliness

According to the Evolutionary Theory of Loneliness, consumers with state loneliness tend to seek social connections to adapt to the negative impacts of loneliness quickly (Lai et al., 2025). In contrast, those with trait loneliness may avoid social contact due to multiple past social failures (Cacioppo and Cacioppo, 2018). Therefore, when consumers have a higher state of loneliness, they are more likely to find social connections to alleviate loneliness. Virtual agents with natural responses may convey greater social warmth and conversational engagement, thereby fostering a stronger sense of social connection (Mourey et al., 2017). Such perceived social warmth and affinity may reduce psychological distance, which in turn can help alleviate feelings of loneliness (Dang and Liu, 2023). Consequently, consumers facing virtual agents with natural responses (vs. scripted responses) will more likely use them. Conversely, consumers with trait loneliness tend to avoid social connections and will more likely use virtual agents with scripted responses (vs. natural responses). Based on this, the following hypotheses are proposed:

*H3*: State loneliness positively moderates the effect of response types on consumers' willingness to use. Specifically, under the condition of high state loneliness, consumers facing virtual agents with natural responses (vs. scripted responses) will have a stronger willingness to use.

*H4*: Trait loneliness negatively moderates the effect of response types on consumers' willingness to use. Specifically, under the condition of high trait loneliness, consumers facing virtual agents with scripted responses (vs. natural responses) will have a stronger willingness to use.

While most research indicates that loneliness can lead to lower expectations of others' trust (Sease et al., 2024; Yuan et al., 2022), Bellucci and Park's recent research demonstrates that trusting behaviors tend to run counter to a person's expectations of another's trustworthiness, i.e., lonely people engage in trusting behaviors to a greater extent (Bellucci and Park, 2024). Moreover, loneliness is a fundamentally affective state (Sease et al., 2024), suggesting that it may be more closely associated with affective trust than with cognitive trust. Building on the previous hypotheses, we speculate that for consumers with high state loneliness who desire social interaction, emotional connections formed with virtual agents with natural responses may positively influence their affective trust, leading to a higher willingness to use these virtual agents than those with scripted responses. Conversely, for consumers who exhibit a high level of trait loneliness and tend to avoid social interactions, emotional connections with virtual agents with natural responses may instead undermine their affective trust, resulting in a lower willingness to engage with these virtual agents. Based on this, the following hypotheses are proposed:

*H5*: State loneliness positively moderates the impact of response types on affective trust and willingness of consumers to use. Specifically, consumers facing virtual agents with natural responses (vs. scripted responses) will exhibit higher affective trust under high state loneliness, leading to a higher willingness to use.

*H6*: Trait loneliness negatively moderates the impact of response types on affective trust and willingness of consumers to use. Specifically, consumers facing virtual agents with scripted responses (vs. natural responses) will exhibit higher affective trust under high trait loneliness, leading to a higher willingness to use.

**Figure 1:**
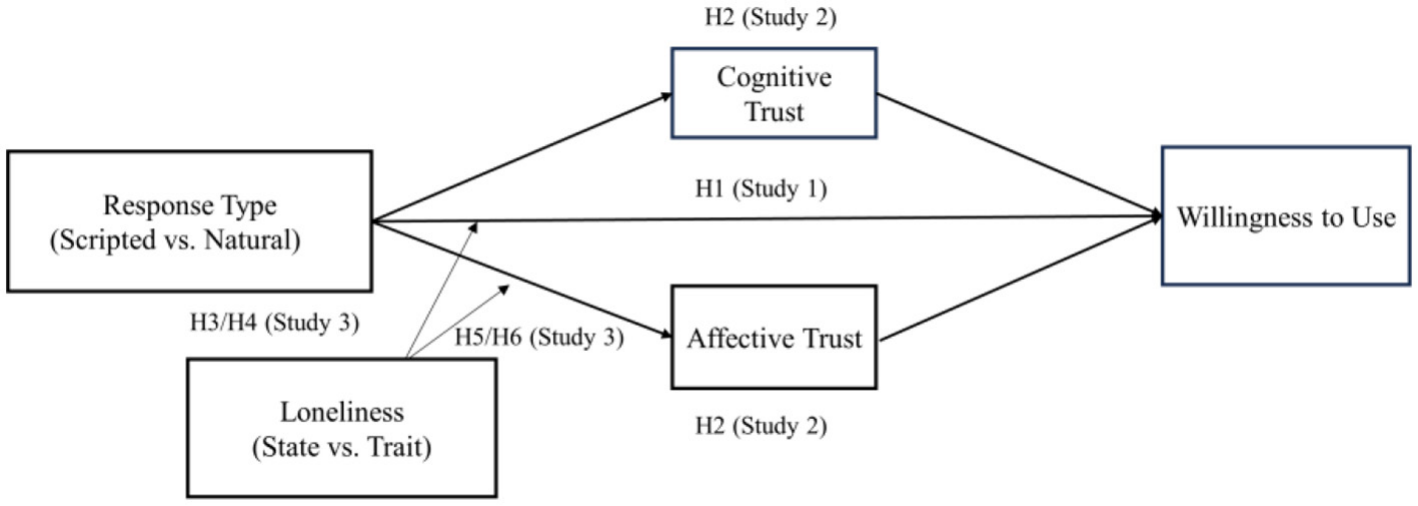
Conceptual model.

The conceptual model is shown in Figure 1.

## 4 Materials and methods

### 4.1 Pre-experiment

To test the success of manipulating the response type (scripted vs. natural), we conducted a pre-experiment.

#### 4.1.1 Stimulus design

To enhance the participants' sense of presence, we abandoned the use of pictures and text as experimental stimuli and instead used the Tencent (Shenzhen, China) to create videos of conversations between consumers and virtual agents in an AI customer service setting. These videos featured virtual agents with high form realism and different response types. The virtual agents in the videos demonstrated the ability to interact with consumers in real-time through text and voice, with appropriate and real-time hand and facial movements, making it easier for participants to understand the concept and application scenarios of the virtual agents. Based on the research of Kim et al. (2024), we designed two response types of virtual agents for dialogue: scripted and natural. Virtual agents with scripted responses provide short, mechanical responses during interactions, while virtual agents with natural responses provide more personalized, in-depth responses (Kim et al., 2024).

#### 4.1.2 Participants

We recruited 100 participants from Credamo (Beijing, China) (59.2% female; aged 18–65) to participate in a between-subjects experiment with one factor (response type: scripted vs. natural). Credamo is a popular online questionnaire collection platform used by Chinese scholars, and the data collected have been adopted and published in several management journals, such as Journal of Marketing, Journal of Business Research, Computers in Human Behavior, and so on. Participants must have experience interacting with AI-driven conversational virtual agents (such as customer service chatbots) to ensure their familiarity with virtual agent interactions. Before the questionnaire begins, participants are asked to answer, “Have you ever interacted with a virtual assistant or chatbot (e.g., for customer service, information retrieval, or entertainment)?” Those who answered “no” were excluded, ensuring that participants could objectively evaluate virtual agent behavior based on their own experience, avoiding biases caused by the novelty effect. We then excluded two participants who failed the attention test, which involved selecting the number 1 in a specific question within the questionnaire. A total of 98 participants met the criteria and were included in the study (59.2% female; aged 18–65 years), and each participant was given a monetary compensation of 1 RMB.

#### 4.1.3 Procedures and measurements

First, participants were divided into two equal groups (scripted: 49 people; natural: 49 people) and invited to watch a video of a virtual agent engaging in a conversation with a smart customer service representative as either a scripted or natural type of response. They were then asked to imagine themselves as the customer in the conversation. See Appendix A for images and text from the video, as well as the web link. Then, each participant was asked to complete two questions about the response types: “Is the verbal response of the virtual agent in the video scripted?” and “Is the verbal response of the virtual agent in the video natural?” (1 = Strongly disagree, 7 = Strongly agree). The questions were adapted from Kim et al. (2024) on the scale of behavioral realism, with the addition of a reverse-coded item, “The language responses of the virtual agents in the video are scripted.” The two items exhibited a significant negative inter-item correlation (*r* = −0.68^**^), consistent with the reverse-coded design. And the Spearman–Brown coefficient was 0.81, indicating good reliability. Because Cronbach's alpha is not appropriate for assessing the reliability of a two-item scale, reliability was evaluated using the inter-item correlation and the Spearman–Brown coefficient, which are commonly recommended for two-item measures. This also applies to subsequent experiments.

#### 4.1.4 Results

Since the normality test results showed that the reaction type scores of the virtual agents did not conform to a normal distribution (*p* < 0.05), given the ordinal nature of the Likert-type scale and the observed non-normal distribution, we employed the Mann–Whitney U-test for group comparisons. We converted the “scripted” (reverse-encoded) scores and combined them with the “natural” scores to form an average value, which we used as the virtual agent's behavioral realism score. The results showed that the virtual agents with scripted responses had lower scores compared to the virtual agents with natural responses (*M*_Scripted_ = 3.45, SD_Scripted_ = 1.03; *M*_Natural_ = 4.67, SD_Natural_ = 0.69, *Z* = −5.90, *p* < 0.001, with a large effect size: *r* = 0.60). These results indicate that the virtual agent's response type (scripted vs. natural) was successfully manipulated.

### 4.2 Study 1

Study 1 used a one-factor (response types: scripted vs. natural) between-subjects experimental design to validate the effect of the response types of virtual agents (scripted vs. natural) on consumers' willingness to use them.

#### 4.2.1 Participants

This experiment recruited 107 participants from Credamo. Before the questionnaire began, participants were asked to answer the question, “Have you ever interacted with a virtual assistant or chatbot (e.g., for customer service, information retrieval, or entertainment)?” (Yes/No). Respondents who answered “No” were screened out. We then excluded 12 participants who failed the attention test, which involved selecting the number 1 in a specific question within the questionnaire. Ninety-five participants met the standards and were included in the study (67.37% female, 18–65 years old), and each was given a monetary compensation of 1 RMB. Before the start of the experiment, participants were required to answer a question about whether they had used virtual agents, and those who did not meet the requirements were screened out. Studies have reported that most users of AI-supported virtual agents are young, middle-aged, or highly educated Internet users (Wang C. et al., 2024), and the demographic characteristics of our participants are consistent with this (see Table 1).

**Table 1:** Demographic characteristics of participants.

**Characteristics**		**Study 1 (*N* = 95)**	**Study 2 (*N* = 148)**	**Study 3 (*N* = 219)**
		**Frequency**		
Gender	Male	31	60	80
	Female	64	88	139
Age	Under 18 years old	1	0	0
	18–25 years old	25	39	46
	26–35 years old	30	67	107
	36–45 years old	23	30	50
	46–55 years old	5	10	14
	56 years old or older	1	2	2
Education	Less than high school	0	0	1
	High school	5	7	3
	University/college	73	110	162
	Master and above	17	31	53
Monthly income	Less than RMB 1,000	2	1	2
	RMB 1,001–2,000	6	13	11
	RMB 2,001–5,000	21	30	32
	RMB 5,001–10,000	32	53	84
	More than RMB 10,000	34	51	90

#### 4.2.2 Procedure

First, participants were randomly divided into two groups (scripted: 47 people; natural: 48 people). Then, participants were informed that they had been invited to assist in a study on virtual agents. Next, they are presented with a textual description of the virtual agent and pictures to help participants further understand it. We provided definitions of both to make it easier for participants to understand the two response types. We then showed the participants a video of a virtual agent of one of the response types as a dialog between an intelligent customer service agent and a consumer. Participants were then invited to watch a video in which a virtual agent with one of the response types interacted with consumers as an intelligent customer service agent, and to imagine themselves as the consumer in the conversation. A female figure was chosen for the virtual agent in the video. See Appendix A for images and text from the video, as well as the web link.

#### 4.2.3 Measurement

Building on prior literature, we measured the validity of the manipulation of response types by participants answering the following questions: “Is the verbal response of the virtual agent in the video scripted?”; “Is the verbal response of the virtual agent in the video natural?” (Kim et al., 2024) (*r* = −0.70^**^, Spearman–Brown coefficient = 0.82). Participants also indicated their willingness to use virtual agents by answering three questions (Xie and Lei, 2022) (α = 0.84). A Likert scale was used for all measures (1 = Strongly disagree, 7 = Strongly agree). See Appendix B for detailed scales.

#### 4.2.4 Results

*Validity and reliability*: We report the CR and AVE to further test the reliability and validity of the scale in Table 2. And the VIF values were reported in Table 3 to test for multicollinearity.

**Table 2:** Combined reliability and average variance extractions.

**Constructs and items**	**Study 1**			**Study 2**			**Study 3**		
	**Factor loading**	**CR**	**AVE**	**Factor loading**	**CR**	**AVE**	**Factor loading**	**CR**	**AVE**
Willingness to use		0.91	0.76		0.91	0.77		0.92	0.79
WU1	0.869			0.862			0.890		
WU2	0.845			0.874			0.875		
WU3	0.902			0.892			0.896		
Cognitive trust					0.93	0.57		0.96	0.71
C1				0.740			0.734		
C2				0.736			0.764		
C3				0.672			0.858		
C4				0.646			0.769		
C5				0.804			0.894		
C6				0.793			0.892		
C7				0.855			0.893		
C8				0.795			0.849		
C9				0.800			0.879		
C10				0.740			0.855		
C11				0.675			0.859		
Trait Loneliness								0.91	0.50
T1							0.735		
T2							0.658		
T3							0.771		
T4							0.688		
T5							0.736		
T6							0.631		
T7							0.705		
T8							0.574		
T9							0.776		
T10							0.771		

**Table 3:** Multicollinearity in the Study 1, Study 2, and Study 3 (VIF).

**Variables**	**Study 1**	**Study 2**	**Study 3**
Response type	1.00	2.23	2.47
Cognitive trust		2.20	2.03
Affective trust		2.63	2.84
State loneliness			1.13
Trait loneliness			1.58

*Manipulation test*: First, we tested whether the virtual agent response type scores conformed to a normal distribution, and the normality test showed that the data did not conform to a normal distribution (*p* < 0.05). After that, we analyzed the response type scores of the virtual agents using the Mann-Whitney U-test, which showed that the virtual agents with scripted responses had lower scores compared to the virtual agent with natural responses (*M*_scripted_ = 2.51, SD_scripted_ = 0.83; *M*_natural_ = 4.17, SD_natural_ = 0.97, *Z* = −6.80, *p* < 0.001, with a large effect size: *r* = 0.70), suggesting that the virtual agent's response types (scripted vs. natural) manipulation was successful.

**Figure 2:**
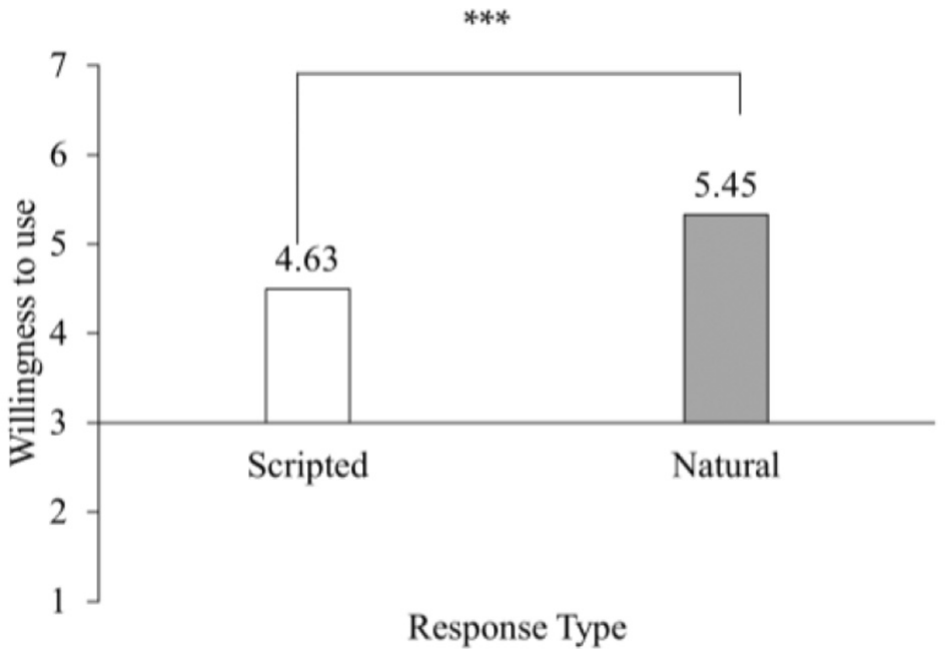
Willingness to use as a function of response type in Study 1. *N* = 95. ***p < 0.001.

*Main effect*: Since the normality test showed that consumers' willingness to use did not conform to a normal distribution (*p* < 0.05), we analyzed consumers' willingness to use using the Mann-Whitney U-test, and the results are shown in Figure 2. The willingness of consumers to use is higher under the condition of natural responses than under the condition of scripted responses (*M*_Scripted_ = 4.63, SD_Scripted_ = 1.26; *M*_Natural_ = 5.45, SD_Natural_ = 1.03, *Z* = −3.51, *p* < 0.001, indicating a medium-to-large effect size: *r* = 0.36), which confirms H1.

### 4.3 Study 2

This study also utilized a one-factor (response types: scripted vs. natural) between-subjects experimental design to validate the mediating roles of cognitive trust and affective trust of consumers. A male virtual agent was used in this study to eliminate the potential interference of virtual agent gender.

#### 4.3.1 Participants

This experiment recruited 164 participants from Credamo. Before the questionnaire began, participants were asked to answer the question, “Have you ever interacted with a virtual assistant or chatbot (e.g., for customer service, information retrieval, or entertainment)?” (Yes/No). Respondents who answered “No” were screened out. We then excluded 16 participants who failed the attention screening test, which involved selecting the number 1 in a specific question within the questionnaire. Eventually, 148 participants met the standards and were selected for the study (59.46% female, 18–65 years), each given a monetary compensation of 1 RMB. Demographic information is presented in Table 1.

#### 4.3.2 Procedure

Like Study 1, participants were divided into two groups (scripted: 73 people; natural: 75 people). Next, they were presented with a textual introduction and pictures of an virtual agent to help participants understand it. We provided both definitions to facilitate participants' understanding of the two response types. We showed a video of a virtual agent of one of the response types as a dialog between an intelligent customer service agent and a consumer. Unlike Study 1, the virtual agent of the video was male, which increased the robustness of the study. See Appendix A for images and text from the video, as well as the web link.

#### 4.3.3 Measurement

Afterward, participants rated the response type (*r* = −0.82^**^, Spearman–Brown coefficient = 0.90) and willingness to use (α = 0.85), respectively. These measurement scales were like those in Study 1. In addition, we measured participants' cognitive trust in the virtual agents with 11 items (α = 0.87), and two items measured affective trust (*r* = 0.55^**^, Spearman–Brown coefficient = 0.71) (Wang et al., 2016). Cognitive trust includes three dimensions—competence, benevolence, and integrity—while affective trust (such as warmth and caring) is viewed as a single construct; the questionnaire items on cognitive trust are more than affective trust (Wang et al., 2016). All measures were taken using a Likert scale (1 = Strongly disagree, 7 = Strongly agree). See Appendix B for detailed scales.

#### 4.3.4 Results

*Validity and reliability testing*: We report the CR and AVE to further test the reliability and validity of the scale in Table 2. And the VIF values were reported in Table 3 to test for multicollinearity.

*Manipulation test*: First, we tested whether the virtual agent response type scores conformed to a normal distribution, and the normality test showed that the data did not conform to a normal distribution (*p* < 0.05). After that, we analyzed the response type scores of the virtual agents using the Mann-Whitney U-test, which showed that the virtual agents with scripted responses had lower scores compared to the virtual agent with natural responses (*M*_scripted_ = 3.21, SD_scripted_ = 0.80; *M*_natural_ = 4.95, SD_natural_ = 0.78, *Z* = −9.60, *p* < 0.001, with a large effect size: *r* = 0.79), suggesting that the virtual agent's response types (scripted vs. natural) were successfully manipulated.

**Figure 3:**
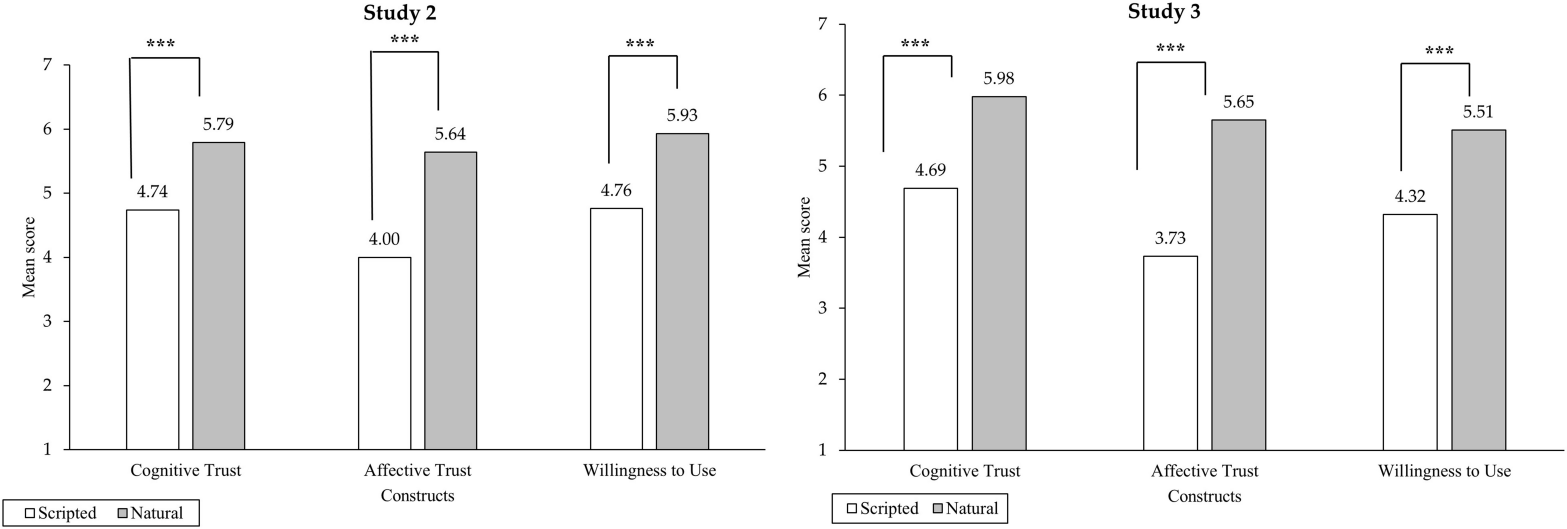
Mean perceived cognitive trust, affective trust, and willingness to use as a function of response type in Study 2 and Study 3. Nstudy2 = 148; Nstudy3 = 219. ***p < 0.001.

*Main effect*: Since the normality test showed that consumers' willingness to use did not conform to a normal distribution (*p* < 0.05), we analyzed consumers' willingness to use using the Mann-Whitney U-test, and the results are shown in Figure 3. The willingness of consumers to use is higher under the condition of natural responses than under the condition of scripted responses (*M*_Scripted_ = 4.76, SD_Scripted_ = 0.87; *M*_Natural_ = 5.93, SD_Natural_ = 0.62, *Z* = −7.96, *p* < 0.001, with a large effect size: *r* = 0.65), which confirms H1.

**Figure 4:**
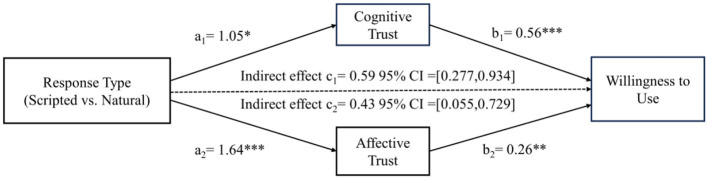
The mediating test in Study 2. *p < 0.05, **p < 0.01, ***p < 0.001.

*Mediation effect*: We conducted the following study using Model 4 for bootstrap analysis to examine the mediating role of consumers' cognitive and affective trust in virtual agents (Preacher and Hayes, 2008). The findings revealed significant mediating effects of cognitive and affective trust [β_cognitivetrust_ = 0.59, 95% CI = (0.277, 0.935); β_affectivetrust_ = 0.43, 95% CI = (0.055, 0.729)]. These results supported H2. Specifically, there was a significant effect of the virtual agent's response types on cognitive trust (β = 1.05, *p* < 0.001) and affective trust (β = 1.64, *p* < 0.001). Cognitive trust (β = 0.56, *p* < 0.001) and affective trust (β = 0.26, *p* < 0.01) had a significant impact on willingness to use. However, the direct impact was non-significant [95% CI = (−0.190, 0.490)], indicating that the direct effect became non-significant after accounting for the indirect effects via cognitive and affective trust (see Figure 4).

### 4.4 Study 3

This study examined the mediating role of consumer affective trust and the moderating role of state loneliness and trait loneliness through a moderated mediation design, using a one-factor (response types: scripted vs. natural) between-subjects experimental design.

#### 4.4.1 Participants

This experiment recruited 227 participants from Credamo. Before starting the questionnaire, participants were asked to answer the question, “Have you ever interacted with a virtual assistant or chatbot (e.g., for customer service, information retrieval, or entertainment, Yes/No). Respondents who answered “No” were screened out. We then excluded 8 participants who failed the attention test, which involved selecting the number 1 in a specific question within the questionnaire. Eventually, 219 participants met the standards and were selected for the study (63.47% female, 18–65 years old) and were given a monetary compensation of 2 RMB each. Demographic information is presented in Table 1.

#### 4.4.2 Procedure

Like Studies 1 and 2, people were randomly assigned to two groups (scripted: 105 people; natural: 110 people) and were first informed that they had been invited to assist in a study on virtual agents. Next, they were presented with a textual introduction and pictures of a virtual agent to help participants understand it. We provided both definitions to facilitate participants' understanding of the two response types. We showed a video of a virtual agent of one of the response types as a dialog between an intelligent customer service agent and a consumer. The virtual agents of the video were the same as in Study 1. See Appendix A for images and text from the video, as well as the web link.

#### 4.4.3 Measurement

Afterward, participants rated the response type (*r* = −0.83^**^, Spearman–Brown coefficient = 0.91), willingness to use (α = 0.86), cognitive trust (α = 0.94), and affective trust (*r* = 0.75^**^, Spearman–Brown coefficient = 0.85), respectively. These measurement scales were similar to Study 1 and 2. Additionally, we measured participants' trait loneliness using 10 items on a 5-point Likert scale (1 = never, 5 = often) (α = 0.74) (Pieters, 2013). After reverse coding items with positive wording, the mean was calculated as the trait loneliness score, with higher scores indicating greater loneliness. See Appendix B for detailed scales. We also used participants to rate their current state loneliness on a visual analog scale (VAS), which was operationalized as participants rated from 0 (“not lonely at all”) to 100 (“very lonely”) (Berger et al., 2024).

#### 4.4.4 Results

*Validity and reliability testing*: We report the CR and AVE to further test the reliability and validity of the scale in Table 2. And the VIF values were reported in Table 3 to test for multicollinearity.

*Manipulation test*: First, we tested whether the virtual agent response type scores conformed to a normal distribution, and the normality test showed that the data did not conform to a normal distribution (*p* < 0.05). After that, we analyzed the response type scores of the virtual agents using the Mann-Whitney U-test, which showed that the virtual agents with scripted responses had lower scores compared to the virtual agent with natural responses (*M*_scripted_ = 3.23, SD_scripted_ = 1.02; *M*_natural_ = 5.39, SD_natural_ = 0.88, *Z* = −11.57, *p* < 0.001, with a large effect size: *r* = 0.78), suggesting that the virtual agent's response type (scripted vs. natural) were successfully manipulated.

*Main effect*: Since the normality test showed that consumers' willingness to use did not conform to a normal distribution (*p* < 0.05), we analyzed consumers' willingness to use using the Mann-Whitney U-test, and the results are shown in Figure 3. The willingness of consumers to use is higher under the condition of natural responses than under the condition of scripted responses (*M*_Scripted_ = 4.32, SD_Scripted_ = 0.95; *M*_Natural_ = 5.51, SD_Natural_ = 1.02, *Z* = −8.38, *p* < 0.001, with a large effect size: *r* = 0.57), which confirms H1.

**Figure 5:**
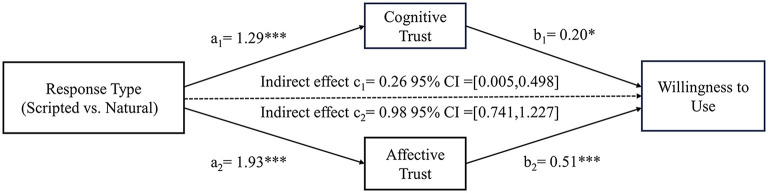
The mediating test by bootstrap analysis in Study 3. *p < 0.05, ***p < 0.001.

*Mediation effect*: We conducted the following study using Model 4 for bootstrap analysis to examine the mediating role of consumers' cognitive and affective trust in virtual agents (Preacher and Hayes, 2008). The findings revealed significant mediating effects of cognitive and affective trust [β_cognitivetrust_ = 0.26, 95% CI = (0.053, 0.498); β_affectivetrust_ = 0.98, 95% CI = (0.741, 1.227)]. These results supported H2. Specifically, there was a significant effect of the virtual agent's response types on cognitive trust (β = 1.29, *p* < 0.001) and affective trust (β = 1.93, *p* < 0.001). Cognitive trust (β = 0.20, *p* < 0.05) and affective trust (β = 0.51, *p* < 0.001) had a significant impact on willingness to use. However, the direct impact was non-significant [95% CI = (−0.407, 0.291)], indicating that the direct effect became non-significant after accounting for the indirect effects via cognitive and affective trust (see Figure 5).

**Figure 6:**
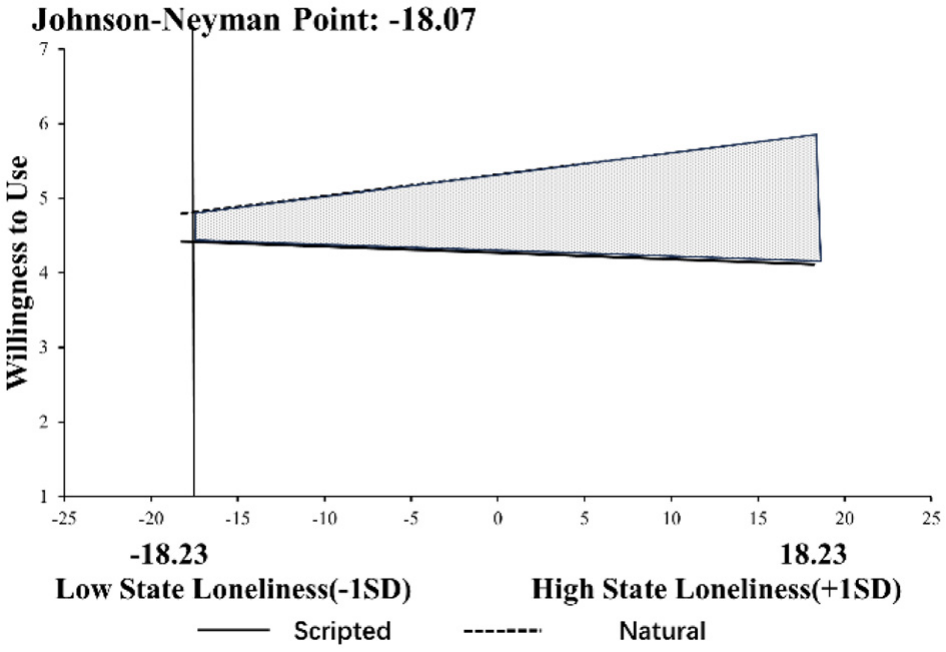
Johnson–Neyman's interaction plot for the willingness to use for state loneliness in Study 3.

*Modulatory effect*: Both trait loneliness (*M* = 2.62, SD = 0.58) and state loneliness (*M* = 49.10, SD = 18.23) exhibit sufficient variability. We utilized Process Model 1 (Hayes, 2017) to explore the moderating effect of state loneliness on consumers' intention to use. The results show that the interaction of response type and state loneliness (β = 0.04, *t* = 4.993, *p* < 0.001) significantly affects consumers' willingness to use. Importantly, the inclusion of the interaction term led to a meaningful increase in explained variance (Δ*R*^2^ = 0.074, *F* = 24.93, *p* < 0.001), suggesting a substantial moderating effect of state loneliness. Since this experiment's moderating variable (state loneliness) is continuous, it was combined with the Johnson-Neyman method for Floodlight analysis (Spiller et al., 2013), shown in Figure 6. The results indicated that when state loneliness exceeded a mean-centered value of −18.07, the conditional effect of response type on willingness to use became significant (β = 0.38, *t* = 1.971, *p* = 0.05). Specifically, among consumers with high state loneliness, virtual agents with natural responses triggered more willingness to use compared to virtual agents with scripted responses. These results support H3.

**Figure 7:**
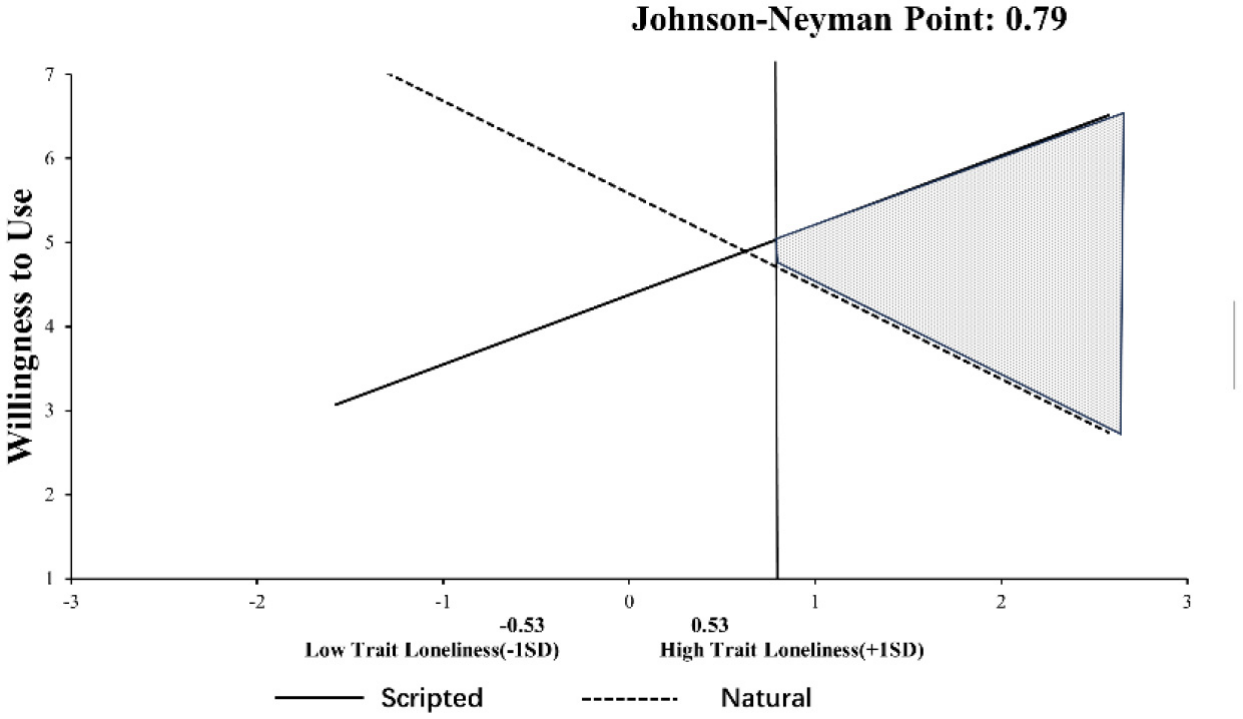
Johnson–Neyman's interaction plot for the willingness to use for trait loneliness in Study 3.

We utilized Process Model 1 (Hayes, 2017) to explore the moderating effect of trait loneliness on consumers' intention to use. The results show that the interaction of response type and trait loneliness (β = −2.05, *t* = −9.497, *p* < 0.001) significantly affects consumers' willingness to use. Importantly, the inclusion of the interaction term led to a substantial increase in explained variance (Δ*R*^2^ = 0.217, *F* = 90.19, *p* < 0.001), suggesting a strong moderating effect of trait loneliness. Since this experiment's moderating variable (trait loneliness) is continuous, it was combined with the Johnson-Neyman method for Floodlight analysis (Spiller et al., 2013), shown in Figure 7. The results showed that when trait loneliness exceeded a mean-centered value of 0.79, the conditional effect of response type became significantly negative (β = −0.40, *t* = −1.971, *p* = 0.05). Specifically, among consumers with high trait loneliness, virtual agents with scripted responses triggered more willingness to use compared to virtual agents with natural responses. These results support H4.

We explored the relationship between state loneliness and consumer affective trust using Process Model 1 (Hayes, 2017), aiming to verify the moderating effect of state loneliness on affective trust. Results showed that the interaction between response type and state loneliness (β = 0.02, *t* = 3.167, *p* < 0.05) had a significant effect on consumer affective trust.

**Figure 8:**
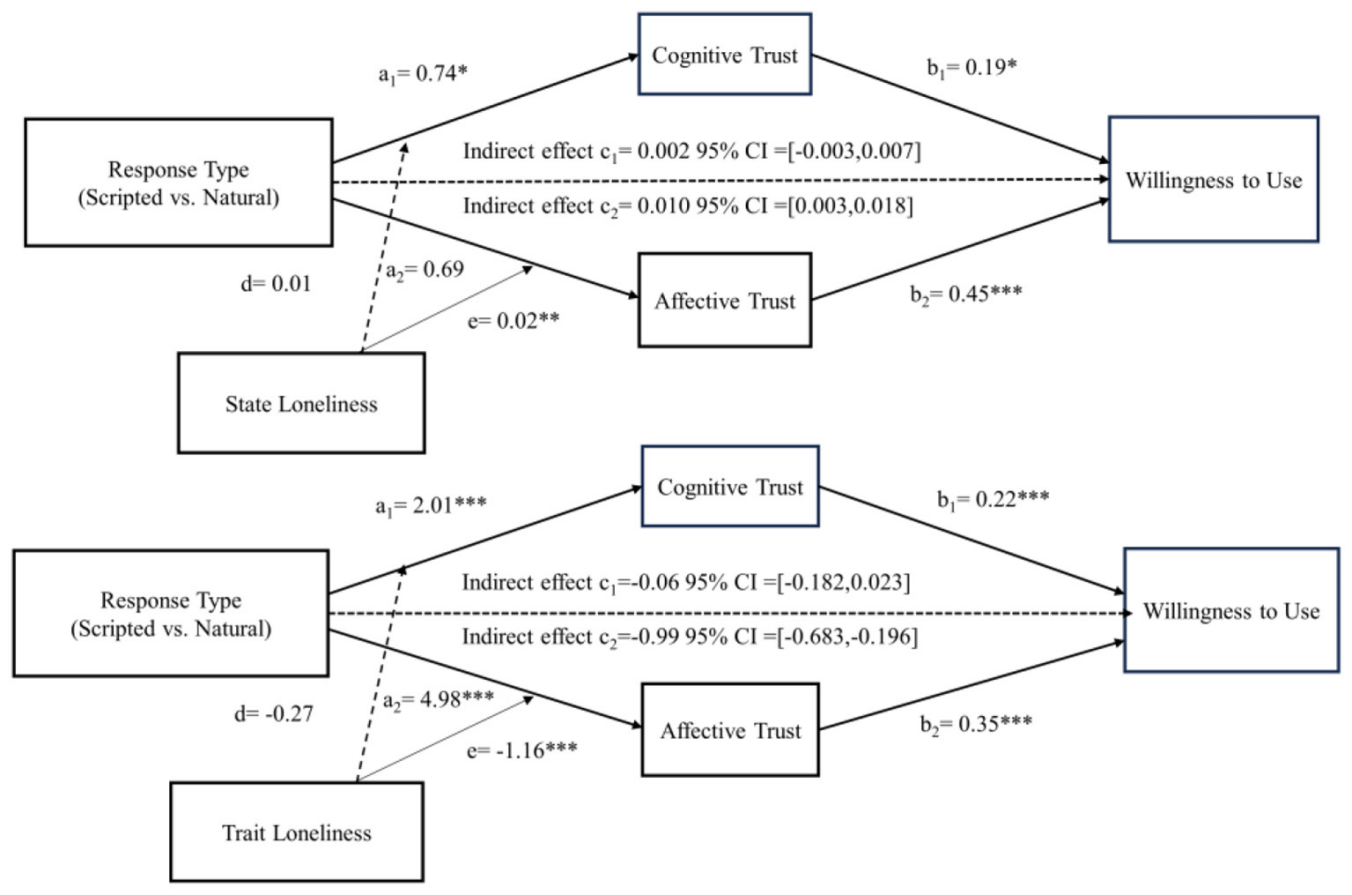
The moderated-mediation effect test of state and trait loneliness in Study 3. *p < 0.05, **p < 0.01, ***p < 0.001.

To further examine the proposed moderated mediation mechanism, we conducted analyses using PROCESS Model 8 (Preacher and Hayes, 2008). The index of moderated mediation was statistically significant [index = 0.010, 95% CI (0.003, 0.018)], excluding zero, which indicates a meaningful conditional indirect effect. The interaction between the response types and state loneliness strongly predicted consumers' affective trust in virtual agents (β = 0.02, *t* = 3.167, *p* < 0.01). It did not predict substantial cognitive trust in virtual agents (β = 0.01, *t* = 1.816, *p* > 0.05). Moreover, when affective trust was included in the model, the direct effect of response type on willingness to use became non-significant [95% CI = (−0.305, 0.388)], indicating that the indirect effect via affective trust accounted for the relationship. These results provide support for H5 (see Figure 8).

We explored the relationship between trait loneliness and consumer affective trust using Process Model 1 (Hayes, 2017), aiming to verify the moderating effect of trait loneliness on affective trust. Results showed that the impact of response type (β = 4.98, *t* = 8.504, *p* < 0.001), trait loneliness (β = 1.74, *t* = 5.277, *p* < 0.001), and their interaction (β = −1.16, *t* = −5.336, *p* < 0.001) on consumer affective trust were all significant.

Using PROCESS Model 8 (Preacher and Hayes, 2008), we further tested the moderated mediation model. The index of moderated mediation was significant [index = −0.406, 95% CI (−0.683, −0.196)], indicating a substantial conditional indirect effect through affective trust. The interaction between the response type of virtual agents and trait loneliness significantly and negatively affected consumers' affective trust in virtual agents (β = −1.16, *t* = −5.336, *p* < 0.001). It did not predict substantial cognitive trust in virtual agents (β = −0.27, *t* = −1.443, *p* > 0.05). These results indicate that affective trust serves as the primary pathway through which response type influences willingness to use among consumers with varying levels of trait loneliness, providing support for H6 (see Figure 8). The results of the moderated mediation effect tests in Study 3 are shown in Appendix C.

## 5 Discussion

### 5.1 Key findings

The main objective of this study is to examine how lonely consumers respond to different response types of virtual agents and to explore the underlying mechanisms. The key findings can be summarized as follows:

First, the results indicate that virtual agents with a higher level of behavioral realism—manifested through natural (as opposed to scripted) responses—are significantly associated with greater consumer willingness to use. Specifically, when virtual agents exhibit natural responses, compared with scripted responses, they can inspire higher cognitive trust and affective trust in consumers, and these two types of trust are in turn associated with increased willingness to use.

Upon closer examination of lonely consumers, a different story emerged. Specifically, consumers with high state loneliness showed greater willingness to use virtual agents with natural responses. In contrast, consumers with high trait loneliness showed greater willingness to use virtual agents with scripted responses. Further analyses suggest that these differences may be explained by the distinct associations between types of loneliness and trust in human–AI interactions. For consumers with high state loneliness, virtual agents with natural responses were positively associated with their affective trust. In contrast, for consumers with high trait loneliness, virtual agents with natural responses were negatively related to affective trust, which was in turn linked to lower willingness to use.

### 5.2 Theoretical contribution

First, this study contributes to the existing literature on behavioral realism in virtual agents by examining how different response types are associated with users' willingness to use virtual agents. Prior research has focused on the impact of other aspects of behavioral realism on customer relationships and behavioral intentions, such as reciprocity and self-disclosure behaviors, emotional expressions, and social cues (Lee and Choi, 2017; Puertas et al., 2024; Xie et al., 2024; Yu et al., 2024). In addition, the role of response type as a manifestation of behavioral realism has primarily been examined in the context of virtual influencers, particularly in relation to consumers' willingness to follow (Kim et al., 2024). However, empirical research examining how response types influence consumers' willingness to use virtual agents in conversational settings, particularly in the context of intelligent customer service, remains limited. By focusing on scripted vs. natural responses, this study extends prior research by examining response type as a distinct manifestation of behavioral realism in virtual agent–consumer interactions. Moreover, the findings suggest that cognitive trust and affective trust serve as important explanatory mechanisms linking response types to consumers' willingness to use.

Secondly, by examining heterogeneity among lonely consumers, this study helps clarify previously mixed findings in the literature on loneliness and virtual agents and extends research on loneliness within the human–AI interaction context, thereby enriching applications of the CASA paradigm. Prior research has reported inconsistent findings regarding the role of loneliness in human–AI interactions, with some studies suggesting a positive association between loneliness and willingness to use virtual agents (Bott et al., 2019), while others indicate a negative association (Yu and Fan, 2024). Drawing on psychological research, this study distinguishes between state loneliness and trait loneliness as two analytically distinct forms of loneliness (Jones et al., 1990). The results show that among consumers experiencing higher levels of state loneliness, natural responses are associated with greater willingness to use virtual agents than scripted responses. In contrast, among consumers with high trait loneliness, more human-like response patterns are associated with lower willingness to use virtual agents.

Finally, this study contributes to research on trust in human–AI interactions by examining the association between human–AI trust and loneliness. Prior studies have primarily emphasized the negative association between loneliness and consumer trust. However, this research differentiates between types of loneliness and shows that state and trait loneliness are associated with different patterns of affective trust toward virtual agents. This distinction advances understanding of how consumer loneliness relates to affective trust in human–AI interactions and adds nuance to the existing literature.

### 5.3 Practical implications

Our findings indicate that individuals with high state loneliness may exhibit a stronger inclination toward virtual agents with natural responses, which offer richer social cues and interaction experiences. In contrast, individuals with high trait loneliness may respond more favorably to virtual agents with scripted responses, which involve lower levels of social and emotional engagement. Based on these findings, differentiated virtual agent design strategies could be considered for users experiencing different types of loneliness, with the aim of improving user experience. For people with state loneliness (e.g., temporary social isolation), virtual agents emphasizing emotional expressiveness and relational cues may be more suitable. Generative AI technologies could be used to develop virtual agents with higher behavioral realism, such as more natural language responses and context-sensitive emotional expressions, which may support short-term emotional support scenarios. For people with trait loneliness, virtual agents characterized by function-oriented and low-intensity interactions may be more appropriate. Structured and predictable dialogue systems may help reduce cognitive and emotional load during interaction, potentially supporting sustained use over time among users with trait loneliness.

Additionally, the findings suggest that for users who respond more positively to natural response styles, the perceived naturalness of virtual agent dialogue design may be an important consideration. Currently, many virtual agent services rely primarily on pre-determined or semi-structured scripts for user interaction. With ongoing advancements in generative AI and large language models, companies may explore integrating these technologies to support more flexible and context-sensitive responses, potentially enhancing the perceived naturalness of virtual agent communication.

Finally, beyond commercial application scenarios, the findings of this study can also provide references for the design of virtual agents in non-commercial fields such as public health and education. In public health settings, virtual agents can be used to deliver informational support, emotional companionship, and preliminary psychological assistance. According to this study, matching the response types of virtual agents to the types of loneliness experienced by users will help enhance users' willingness to use them, thereby improving the quality of public health services. In the field of education, virtual agents are often deployed as tutoring assistants. The findings of this study indicate that variations in the behavioral realism of virtual agents may exert differential impacts on students' trust and willingness to interact, which is particularly important for learners experiencing social isolation. Educational institutions can improve the diversity and effectiveness of digital learning environments by adjusting the response types of virtual agents.

### 5.4 Limitations and future research

This study continues to have some limitations that will hopefully be addressed in future research. First, this study examined response types as an overall manifestation of behavioral realism in virtual agents, without disentangling the specific dimensions embedded within response types, such as perceived warmth, usefulness, or personalization. Future research could decompose response types into finer-grained dimensions and examine how these distinct attributes differentially influence individuals' willingness to use virtual agents.

Second, behavioral realism is an inherently multidimensional construct. According to Miao et al. (2022), behavioral realism can be reflected in multiple dimensions, including communication mode, response type, and social presence. In the present study, we operationalized behavioral realism through a dichotomous distinction between scripted and natural responses, which represents a focused yet simplified approach. Beyond response type, behavioral realism may also involve dimensions such as contextual appropriateness and emotional expressiveness. Future research could adopt more fine-grained manipulations and provide illustrative examples to examine how different dimensions of behavioral realism shape consumers' perceptions and behavioral responses.

Third, prior research suggests that the effects of customer perceptions on behavioral outcomes may vary across different stages of the customer–virtual agent relationship. Across the interaction process, the relationship between consumers and virtual agents may be conceptualized as progressing through three stages: an initial exploratory stage, a relationship-building stage, and a relatively stable maturity stage (Jap and Ganesan, 2000). However, the present study did not explicitly examine whether the effects and underlying mechanisms differ across these relationship stages. Future research could refine this line of inquiry by examining how relationship stages condition the effects of virtual agent characteristics on consumer–virtual agent interactions and willingness to use.

Fourth, this study employed video-based materials as experimental stimuli to manipulate the response types of virtual agents. Video stimuli provide richer contextual information and greater immersion than static images or text, which have been commonly used in prior research. However, video materials may still not fully capture the richness, spontaneity, and bidirectional dynamics of real-world human–AI interactions. As a result, participants' responses may differ from those observed in real-time or longitudinal interaction contexts. Future research could adopt more interactive designs, such as real-time conversations with virtual agents, field experiments, or longitudinal methods, to more accurately reflect the complexity of human–AI interactions.

Fifth, although the compensation standard set in this study was consistent with the common practices of local online survey platforms, the relatively low compensation might have affected participants' motivation and engagement, thereby potentially introducing biases. To mitigate the impact of this issue, attention-check items were incorporated into the study, and participants who failed these checks were excluded. Nevertheless, future research could adopt higher compensation incentives or alternative recruitment strategies to further enhance participants' engagement and data quality.

Lastly, this study relied on data collected exclusively in mainland China, which may limit the generalizability of the findings. At present, research on virtual agents and behavioral realism remains relatively limited overall, and studies that systematically compare different response types (scripted vs. natural) based on samples from diverse populations are even scarcer. Most existing relevant studies have been conducted on samples outside Mainland China, yet cultural backgrounds give rise to variations in people's perceptions of technology use, social expectations, and human-AI interaction. For instance, collectivist cultures place greater emphasis on interpersonal harmony and relational cues in communication, whereas individualist cultures prioritize directness and autonomy. Such cultural differences may affect how people perceive and value the behavioral realism characteristics of virtual agents. Therefore, future research is encouraged to conduct cross-cultural validation to examine whether the observed effects hold in different cultural contexts.

## Data Availability

The raw data supporting the conclusions of this article will be made available by the authors, without undue reservation.

## References

[B1] Adam M. Wessel M. Benlian A. (2021). AI-based chatbots in customer service and their effects on user compliance. *Electron*. Mark. 31, 427–445. doi: 10.1007/s12525-020-00414-7

[B2] Anderson C. M. Martin M. M. (1995). The effects of communication motives, interaction involvement, and loneliness on satisfaction: a model of small groups. Small Group Res. 26, 118–137. doi: 10.1177/1046496495261007

[B3] Bach T. A. Khan A. Hallock H. Beltrão G. Sousa S. (2024). A systematic literature review of user trust in AI-enabled systems: an HCI perspective. Int. J. Human Comput. Interact. 40, 1251–1266. doi: 10.1080/10447318.2022.2138826

[B4] Bansal G. Chamola V. Hussain A. Guizani M. Niyato D. (2024). Transforming conversations with AI-A comprehensive study of ChatGPT. *Cogn*. Comput. 16, 2487–2510. doi: 10.1007/s12559-023-10236-2

[B5] Bateson J. E. Hui M. K. (1992). The ecological validity of photographic slides and videotapes in simulating the service setting. *J. Consum*. Res. 19, 271–281. doi: 10.1086/209301

[B6] Bellucci G. (2020). Positive attitudes and negative expectations in lonely individuals. Sci. Rep. 10:18595. doi: 10.1038/s41598-020-75712-3 33122843 PMC7596507

[B7] Bellucci G. Park S. Q. (2024). Loneliness is associated with more trust but worse trustworthiness expectations. Br. J. Psychol. 115, 641–664. doi: 10.1111/bjop.12713 38807533

[B8] Berger R. Hurlemann R. Shamay-Tsoory S. Kanterman A. Brauser M. Gorni J. . (2024). Oxytocin-augmented modular-based group intervention for loneliness: a proof-of-concept randomized controlled trial. Psychother. Psychosom. 93, 169–180. doi: 10.1159/000538752 38754399

[B9] Bott N. Wexler S. Drury L. Pollak C. Wang V. Scher K. . (2019). A protocol-driven, bedside digital conversational agent to support nurse teams and mitigate risks of hospitalization in older adults: case control pre-post study. *J. Med*. Internet Res. 21:e13440. doi: 10.2196/13440 PMC6913375 31625949

[B10] Brandtzaeg P. B. Følstad A. (2018). Chatbots: changing user needs and motivations. Interactions 25, 38–43. doi: 10.1145/3236669

[B11] Buecker S. Maes M. Denissen J. J. A. Luhmann M. (2020). Loneliness and the big five personality traits: a meta-analysis. Eur. J. Personal. 34, 8–28. doi: 10.1002/per.2229

[B12] Burden D. Savin-Baden M. (2019). Virtual Humans: Today and Tomorrow, 1st Edn. New York: CRC Press.

[B13] Cacioppo J. T. Cacioppo S. (2018). “Loneliness in the modern age: an Evolutionary Theory of Loneliness (ETL),” in Advances in Experimental Social Psychology, ed. J. M. Olson (San Diego: Elsevier Academic Press Inc.), 127–197.

[B14] Cacioppo J. T. Patrick W. (2008). Loneliness: Human Nature and the Need for Social Connection, First Edition. New York: WW Norton and Company.

[B15] Chakraborty D. Kumar Kar A. Patre S. Gupta S. (2024). Enhancing trust in online grocery shopping through generative AI chatbots. *J. Bus*. Res. 180:114737. doi: 10.1016/j.jbusres.2024.114737

[B16] Chang W. Park J. (2024). A comparative study on the effect of ChatGPT recommendation and AI recommender systems on the formation of a consideration set. *J. Retail. Consum*. Serv. 78:103743. doi: 10.1016/j.jretconser.2024.103743

[B17] Chen H. Shao B. Yang X. Kang W. Fan W. (2024). Avatars in live streaming commerce: the influence of anthropomorphism on consumers' willingness to accept virtual live streamers. *Comput. Hum*. Behav. 156:108216. doi: 10.1016/j.chb.2024.108216

[B18] Chi O. H. Chi C. G. Gursoy D. Nunkoo R. (2023). Customers' acceptance of artificially intelligent service robots: the influence of trust and culture. Int. J. Inf. Manag. 70:102623. doi: 10.1016/j.ijinfomgt.2023.102623

[B19] Choung H. David P. Ross A. (2023). Trust in AI and its role in the acceptance of AI technologies. Int. J. Human Comput. Interact. 39, 1727–1739. doi: 10.1080/10447318.2022.2050543

[B20] Crolic C. Thomaz F. Hadi R. Stephen A. T. (2022). Blame the Bot: anthropomorphism and anger in Customer-Chatbot interactions. J. Mark. 86, 132–148. doi: 10.1177/00222429211045687

[B21] Dabiran E. Farivar S. Wang F. Grant G. (2024). Virtually human: anthropomorphism in virtual influencer marketing. *J. Retail. Consum*. Serv. 79:103797. doi: 10.1016/j.jretconser.2024.103797

[B22] Dang J. Liu L. (2023). Do lonely people seek robot companionship? A comparative examination of the Loneliness–Robot anthropomorphism link in the United States and China. *Comput. Hum*. Behav. 141:107637. doi: 10.1016/j.chb.2022.107637

[B23] Edwards C. Edwards A. Stoll B. Lin X. Massey N. (2019). Evaluations of an artificial intelligence instructor's voice: Social Identity Theory in human-robot interactions. Comput. Hum. Behav. 90, 357–362. doi: 10.1016/j.chb.2018.08.027

[B24] Fatima J. K. Khan M. I. Bahmannia S. Chatrath S. K. Dale N. F. Johns R. . (2024). Rapport with a chatbot? The underlying role of anthropomorphism in socio-cognitive perceptions of rapport and e-word of mouth. *J. Retail. Consum*. Serv. 77:103666. doi: 10.1016/j.jretconser.2023.103666

[B25] Fraune M. R. Komatsu T. Preusse H. R. Langlois D. K. Au R. H. Y. Ling K. . (2022). Socially facilitative robots for older adults to alleviate social isolation: a participatory design workshop approach in the US and Japan. Front. Psychol. 13:904019. doi: 10.3389/fpsyg.2022.904019 36337527 PMC9629871

[B26] Gillath O. Ai T. Branicky M. S. Keshmiri S. Davison R. B. Spaulding R. . (2021). Attachment and trust in artificial intelligence. *Comput. Hum*. Behav. 115:106607. doi: 10.1016/j.chb.2020.106607

[B27] Gkinko L. Elbanna A. (2023). Designing trust: the formation of employees' trust in conversational AI in the digital workplace. J. Bus. Res. 158:113707. doi: 10.1016/j.jbusres.2023.113707

[B28] Guo Y. Xu L. Wang C. (2025). Exploring the effect of empathic response and its boundaries in artificial intelligence service recovery. *J. Retail. Consum*. Serv. 82:104065. doi: 10.1016/j.jretconser.2024.104065

[B29] Ham J. Li S. Looi J. Eastin M. S. (2024). Virtual humans as social actors: investigating user perceptions of virtual humans' emotional expression on social media. *Comput. Hum*. Behav. 155:108161. doi: 10.1016/j.chb.2024.108161

[B30] Hayes A. F. (2017). Introduction to Mediation, Moderation, and Conditional Process Analysis: A Regression-Based Approach, Second Edn. New York: Guilford publications.

[B31] Ho S. S. Cheung J. C. (2024). Trust in artificial intelligence, trust in engineers, and news media: factors shaping public perceptions of autonomous drones through UTAUT2. Technol. Soc. 77:102533. doi: 10.1016/j.techsoc.2024.102533

[B32] Hu B. Mao Y. Kim K. J. (2023). How social anxiety leads to problematic use of conversational AI: the roles of loneliness, rumination, and mind perception. Comput. Hum. Behav. 145:107760. doi: 10.1016/j.chb.2023.107760

[B33] Jan I. U. Ji S. Kim C. (2023). What (de) motivates customers to use AI-powered conversational agents for shopping? The extended behavioral reasoning perspective. J. Retail. Consum. Serv. 75:103440. doi: 10.1016/j.jretconser.2023.103440

[B34] Jap S. D. Ganesan S. (2000). Control mechanisms and the relationship life cycle: implications for safeguarding specific investments and developing commitment. *J. Mark*. Res. 37, 227–245. doi: 10.1509/jmkr.37.2.227.18735

[B35] Jiang K. Qin M. Li S. (2022). Chatbots in retail: how do they affect the continued use and purchase intentions of Chinese consumers? *J. Consum*. Behav. 21, 756–772. doi: 10.1002/cb.2034

[B36] Jones W. H. Rose J. Russell D. (1990). “Loneliness and social anxiety,” in Handbook of Social and Evaluation Anxiety (Boston, MA: Springer US), 247–266.

[B37] Khennouche F. Elmir Y. Himeur Y. Djebari N. Amira A. (2024). Revolutionizing generative pre-traineds: insights and challenges in deploying ChatGPT and generative chatbots for FAQs. Expert Syst. Appl. 246:123224. doi: 10.1016/j.eswa.2024.123224

[B38] Kim D. Y. Lee H. K. Chung K. (2023). Avatar-mediated experience in the metaverse: the impact of avatar realism on user-avatar relationship. *J. Retail. Consum*. Serv. 73:103382. doi: 10.1016/j.jretconser.2023.103382

[B39] Kim I. Ki C-. W. Lee H. Kim Y-. K. (2024). Virtual influencer marketing: evaluating the influence of virtual influencers' form realism and behavioral realism on consumer ambivalence and marketing performance. J. Bus. Res. 176:114611. doi: 10.1016/j.jbusres.2024.114611

[B40] Kim J. Giroux M. Lee J. C. (2021). When do you trust AI? The effect of number presentation detail on consumer trust and acceptance of AI recommendations. Psychol. Mark. 38, 1140–1155. doi: 10.1002/mar.21498

[B41] Kim J. Kim J. H. Kim C. Park J. (2023). Decisions with ChatGPT: reexamining choice overload in ChatGPT recommendations. *J. Retail. Consum*. Serv. 75:103494. doi: 10.1016/j.jretconser.2023.103494

[B42] Kim T. Lee O-. K. D. Kang J. (2023). Is it the best for barista robots to serve like humans? A multidimensional anthropomorphism perspective. *Int. J. Hosp*. Manag. 108:103358. doi: 10.1016/j.ijhm.2022.103358

[B43] Kozyreva A. Lorenz-Spreen P. Hertwig R. Lewandowsky S. Herzog S. M. (2021). Public attitudes towards algorithmic personalization and use of personal data online: evidence from Germany, Great Britain, and the United States. Humanit. Soc. Sci. Commun. 8, 1–11. doi: 10.1057/s41599-021-00787-w

[B44] Krämer N. C. Rosenthal-von der Pütten A.M. Hoffmann L. (2015). “Social effects of virtual and robot companions,” in The Handbook of the Psychology of Communication Technology, ed. S. S. Sundar (Hoboken, NJ: John Wiley and Sons, Ltd.), 137–159.

[B45] Lai L. Pan Y. Xu R. Jiang Y. (2025). Depression and the use of conversational AI for companionship among college students: the mediating role of loneliness and the moderating effects of gender and mind perception. Front. Public Health 13:1580826. doi: 10.3389/fpubh.2025.1580826 40520273 PMC12162938

[B46] Le H. T. P. M. Park J. Lee S. (2023). Emotion and trust in virtual service assistant design for effective service recovery. J. Retail. Consum. Serv. 74:103368. doi: 10.1016/j.jretconser.2023.103368

[B47] Lee S. Choi J. (2017). Enhancing user experience with conversational agent for movie recommendation: effects of self-disclosure and reciprocity. Int. J. Hum.-Comput. Stud. 103, 95–105. doi: 10.1016/j.ijhcs.2017.02.005

[B48] Legood A. van der Werff L. Lee A. den Hartog D. van Knippenberg D. (2023). A critical review of the conceptualization, operationalization, and empirical literature on cognition-based and affect-based trust. *J. Manag*. Stud. 60, 495–537. doi: 10.1111/joms.12811

[B49] Liu Q. Ma N. Zhang X. (2025). Can AI-virtual anchors replace human internet celebrities for live streaming sales of products? An emotion theory perspective. *J. Retail. Consum*. Serv. 82:104107. doi: 10.1016/j.jretconser.2024.104107

[B50] Maese E. (2023). Almost a Quarter of the World Feels Lonely [WWW Document]. Gallup.com. Available online at: https://news.gallup.com/opinion/gallup/512618/almost-quarter-world-feels-lonely.aspx (Accessed December 18, 2024).

[B51] McAllister D. J. (1995). Affect-and cognition-based trust as foundations for interpersonal cooperation in organizations. *Acad. Manage*. J. 38, 24–59. doi: 10.2307/256727

[B52] Miao F. Kozlenkova I. V. Wang H. Xie T. Palmatier R. W. (2022). An emerging theory of avatar marketing. J. Mark. 86, 67–90. doi: 10.1177/0022242921996646

[B53] Mourey J. A. Olson J. G. Yoon C. (2017). Products as Pals: engaging with anthropomorphic products mitigates the effects of social exclusion. *J. Consum*. Res. 44, 414–431. doi: 10.1093/jcr/ucx038

[B54] Munnukka J. Talvitie-Lamberg K. Maity D. (2022). Anthropomorphism and social presence in Human-Virtual service assistant interactions: the role of dialog length and attitudes. Comput. Hum. Behav. 135:107343. doi: 10.1016/j.chb.2022.107343

[B55] Nass C. Moon Y. (2000). Machines and mindlessness: social responses to computers. J. Soc. Issues 56, 81–103. doi: 10.1111/0022-4537.00153

[B56] Nass C. Moon Y. Fogg B. J. Reeves B. Dryer D. C. (1995). Can computer personalities be human personalities? Int. J. Hum.-Comput. Stud. 43, 223–239. doi: 10.1006/ijhc.1995.1042

[B57] Nass C. Steuer J. Tauber E. R. (1994). “Computers are social actors,” in Proceedings of the SIGCHI Conference on Human Factors in Computing Systems (Boston, MA: ACM), 72–78.

[B58] Nowak K. L. Fox J. (2018). Avatars and computer-mediated communication: a review of the definitions, uses, and effects of digital representations. *Rev. Commun*. Res. 6, 30–53. doi: 10.12840/issn.2255-4165.2018.06.01.015

[B59] Park J. Yun J. Chang W. (2024). Intention to adopt services by AI avatar: a protection motivation theory perspective. *J. Retail. Consum*. Serv. 80:103929. doi: 10.1016/j.jretconser.2024.103929

[B60] Park Y. J. Jones-Jang S. M. (2023). Surveillance, security, and AI as technological acceptance. AI Soc. 38, 2667–2678. doi: 10.1007/s00146-021-01331-9

[B61] Pieters R. (2013). Bidirectional dynamics of materialism and loneliness: not just a vicious cycle. J. Consum. Res. 40, 615–631. doi: 10.1086/671564

[B62] Pot M. Paulussen T. G. Ruiter R. A. Eekhout I. de Melker H. E. Spoelstra M. E. . (2017). Effectiveness of a web-based tailored intervention with virtual assistants promoting the acceptability of HPV vaccination among mothers of invited girls: randomized controlled trial. J. Med. Internet Res. 19:e312. doi: 10.2196/jmir.7449 28877862 PMC5607435

[B63] Preacher K. J. Hayes A. F. (2008). Asymptotic and resampling strategies for assessing and comparing indirect effects in multiple mediator models. Behav. Res. Methods 40, 879–891. doi: 10.3758/BRM.40.3.879 18697684

[B64] Puertas S. M. Manzano M. D. I. Lopez C. S. Cardoso P. R. (2024). Purchase intentions in a chatbot environment: an examination of the effects of customer experience. Oeconomia Copernic. 15, 145–194. doi: 10.24136/oc.2914

[B65] Qiu L. Benbasat I. (2005). Online consumer trust and live help interfaces: the effects of text-to-speech voice and three-dimensional avatars. Int. J. Hum.-Comput. Interact. 19, 75–94. doi: 10.1207/s15327590ijhc1901_6

[B66] Rajaobelina L. Prom Tep S. Arcand M. Ricard L. (2021). Creepiness: its antecedents and impact on loyalty when interacting with a chatbot. Psychol. Mark. 38, 2339–2356. doi: 10.1002/mar.21548

[B67] Razavi S. Z. Schubert L. K. van Orden K. Ali M. R. Kane B. Hoque E. . (2022). Discourse behavior of older adults interacting with a dialogue agent competent in multiple topics. *ACM Trans. Interact. Intell*. Syst. 12:14. doi: 10.1145/3484510

[B68] Reeves B. Nass C. (1996). The media equation: how people treat computers, television, and new media like real people. Camb. UK 10, 19–36.

[B69] Research Nester (2024). Chatbot Market Size and Share | Forecast Report 2036 [WWW Document]. Res. Nester. Available online at: https://www.researchnester.com/reports/chatbot-market/5567 (Accessed January 15, 2025).

[B70] Saporta N. Scheele D. Lieberz J. Stuhr-Wulff F. Hurlemann R. Shamay-Tsoory S. G. . (2021). Opposing association of situational and chronic loneliness with interpersonal distance. Brain Sci. 11:1135. doi: 10.3390/brainsci11091135 34573157 PMC8471414

[B71] Schuetzler R. M. Giboney J. S. Grimes G. M. Nunamaker J. F. (2018). The influence of conversational agent embodiment and conversational relevance on socially desirable responding. Decis. Support Syst. 114, 94–102. doi: 10.1016/j.dss.2018.08.011

[B72] Sease T. B. Sandoz E. K. Yoke L. Swets J. A. Cox C. R. (2024). Loneliness and relationship well-being: investigating the mediating roles of relationship awareness and distraction among romantic partners. *Behav*. Sci. 14:439. doi: 10.3390/bs14060439 PMC11200782 38920770

[B73] Shahzad M. F. Xu S. An X. Javed I. (2024). Assessing the impact of AI-chatbot service quality on user e-brand loyalty through chatbot user trust, experience and electronic word of mouth. *J. Retail. Consum*. Serv. 79:103867. doi: 10.1016/j.jretconser.2024.103867

[B74] Spiller S. A. Fitzsimons G. J. Lynch J. G. McClelland G. H. (2013). Spotlights, floodlights, and the magic number zero: simple effects tests in moderated regression. J. Mark. Res. 50, 277–288. doi: 10.1509/jmr.12.0420

[B75] Suen H-. Y. Hung K-. E. (2023). Building trust in automatic video interviews using various AI interfaces: tangibility, immediacy, and transparency. *Comput. Hum*. Behav. 143:107713. doi: 10.1016/j.chb.2023.107713

[B76] UneeQ (2022). Meet Eve | A UneeQ digital human AI for Kiehl's [WWW Document]. Available online at: https://www.digitalhumans.com/case-studies/kiehls (Accessed July 15, 2025).

[B77] van Pinxteren M. M. E. Pluymaekers M. Lemmink J. Krispin A. (2023). Effects of communication style on relational outcomes in interactions between customers and embodied conversational agents. Psychol. Mark. 40, 938–953. doi: 10.1002/mar.21792

[B78] van Roekel E. Verhagen M. Engels R. C. M. E. Scholte R. H. J. Cacioppo S. Cacioppo J. T. . (2018). Trait and state levels of loneliness in early and late adolescents: examining the differential reactivity hypothesis. *J. Clin. Child Adolesc*. Psychol. 47, 888–899. doi: 10.1080/15374416.2016.1146993 27191708

[B79] Vanman E. J. Kappas A. (2019). “Danger, Will Robinson!” The challenges of social robots for intergroup relations. Soc. Personal. Psychol. Compass 13:e12489. doi: 10.1111/spc3.12489

[B80] Wang C. Hao L. Guo Y. Xie S. Wu Z. (2024). Report on the Development of Generative Artificial Intelligence Applications (2024) - Internet Development Research, China.

[B81] Wang W. Qiu L. Kim D. Benbasat I. (2016). Effects of rational and social appeals of online recommendation agents on cognition-and affect-based trust. Decis. Support Syst. 86, 48–60. doi: 10.1016/j.dss.2016.03.007

[B82] Wang Y.-F. Chen Y-.C. Chien S-.Y. Wang P-.J. (2024). Citizens' trust in AI-enabled government systems. Inf. Polity 29, 293–312. doi: 10.3233/IP-230065

[B83] Wei Y. Syahrivar J. Simay A. E. (2025). Unveiling the influence of anthropomorphic chatbots on consumer behavioral intentions: evidence from China and Indonesia. J. Res. Interact. Mark. 19, 132–157. doi: 10.1108/JRIM-09-2023-0295

[B84] Xie L. Lei S. (2022). The nonlinear effect of service robot anthropomorphism on customers? usage intention: a privacy calculus perspective. *Int. J. Hosp*. Manag. 107:103312. doi: 10.1016/j.ijhm.2022.103312

[B85] Xie Y. Liang C. Zhou P. Jiang L. (2024). Exploring the influence mechanism of chatbot-expressed humor on service satisfaction in online customer service. J. Retail. Consum. Serv. 76:103599. doi: 10.1016/j.jretconser.2023.103599

[B86] Yan H. Wei Y. Xiong H. (2024). How do initial and interactive social cues increase customers' continuance usage intention of Chatbots? Int. J. Human Comput. Interact. 41, 1–18. doi: 10.1080/10447318.2024.2352928

[B87] Yang D. Zhang J. Sun Y. Huang Z. (2024). Showing usage behavior or not? The effect of virtual influencers' product usage behavior on consumers. *J. Retail. Consum*. Serv. 79:103859. doi: 10.1016/j.jretconser.2024.103859

[B88] Yao Q. Kuai L. Jiang L. (2023). Effects of the anthropomorphic image of intelligent customer service avatars on consumers' willingness to interact after service failures. J. Res. Interact. Mark. 17, 734–753. doi: 10.1108/JRIM-06-2022-0164

[B89] Yu J. Dickinger A. So K. K. F. Egger R. (2024). Artificial intelligence-generated virtual influencer: examining the effects of emotional display on user engagement. J. Retail. Consum. Serv. 76:103560. doi: 10.1016/j.jretconser.2023.103560

[B90] Yu L. Fan X. (2024). Lonely human and dominant robot: similarity versus complementary attraction. Psychol. Mark. 41, 1133–1151. doi: 10.1002/mar.21975

[B91] Yuan Y. Jiang S. Yan S. Chen L. Zhang M. Zhang J. . (2022). The relationship between depression and social avoidance of college students: a moderated mediation model. J. Affect. Disord. 300, 249–254. doi: 10.1016/j.jad.2021.12.119 34979184

[B92] Zhang C. Hu M. Wu W. Kamran F. Wang X. (2024). *Unpacking perceived risks and AI trust influences pre-service teachers' AI* acceptance: a structural equation modeling-based multi-group analysis. Educ. Inf. Technol. 30, 2645–2672. doi: 10.1007/s10639-024-12905-7

[B93] Zhang Y. Wang S. (2023). The influence of anthropomorphic appearance of artificial intelligence products on consumer behavior and brand evaluation under different product types. *J. Retail. Consum*. Serv. 74:103432. doi: 10.1016/j.jretconser.2023.103432

[B94] Zhao Z. Kou Y. (2024). Effects of loneliness on short video addiction among college students: the chain mediating role of social support and physical activity. Front. Public Health 12:1484117. doi: 10.3389/fpubh.2024.1484117 39600403 PMC11588628

