# Supplementary material for: How do consumers perceive virtual agents with behavioral realism? The role of loneliness and trust
Source: Front Psychol. 2026 Feb 26;17:1676234. doi: 10.3389/fpsyg.2026.1676234 (PMC12980403; doi:10.3389/fpsyg.2026.1676234)
Supplement: Supplementary file 1 [file Table_1.docx]

Supplementary Material

# Appendix A： Experiment Materials Used in Studies

## Table A.1 Text of the Conversation in the Video Material

| Virtual agent with scripted responses | Virtual agent with natural responses |
| --- | --- |
| A: Hello, Intelligent Customer Service Assistant is at your service! How can I help you?  C: Suggest a camera that's suitable as a gift!  A: Your question can only be answered if there is a product name. Please enter “I want to buy + the product you are inquiring about”.  C: I want to buy a camera  A: Please select a brand (Options about branding appear)  Canon Nikon FUJI RICOH  SONY DJI Panasonic SONGDIAN  All the above will be fine.  C: Sony  A: Please tell me the price range  C: 3500-4500 RMB  A: Please tell me about other needs  C: Need to be suitable for portraits  A: We found the following products for you. You can click on the product to view specific information (Product images and links appear) | A: Honey, I'm your sales assistant UU! What do we want to buy today?  C: Suggest a camera that is suitable as a gift!  A: What's your budget for a gift? Let me help you.  C: About four thousand dollars  A: Wow, that's an expensive gift. Then you can look at a DSLR camera.  Canon's EOS R100 is a high-quality camera, and SONY's ZV-1F has a wide-angle zoom lens, which is great for vlogging.  These two have been on sale recently, and the price is right too.  C: Which one is good for portraits  A: This one from SONY is more suitable for portraits  C: Okay.  A: Click on the product image, and I can show you more details. (Product images and links appear) |

Note: A means avatar. C means consumer.

## Table A.2 Virtual agent in Video Material

| The Female virtual agent of High Formal Realism in Study 1 and Study 3 | The Male virtual agent of High Formal Realism in Study 2 |
| --- | --- |
| 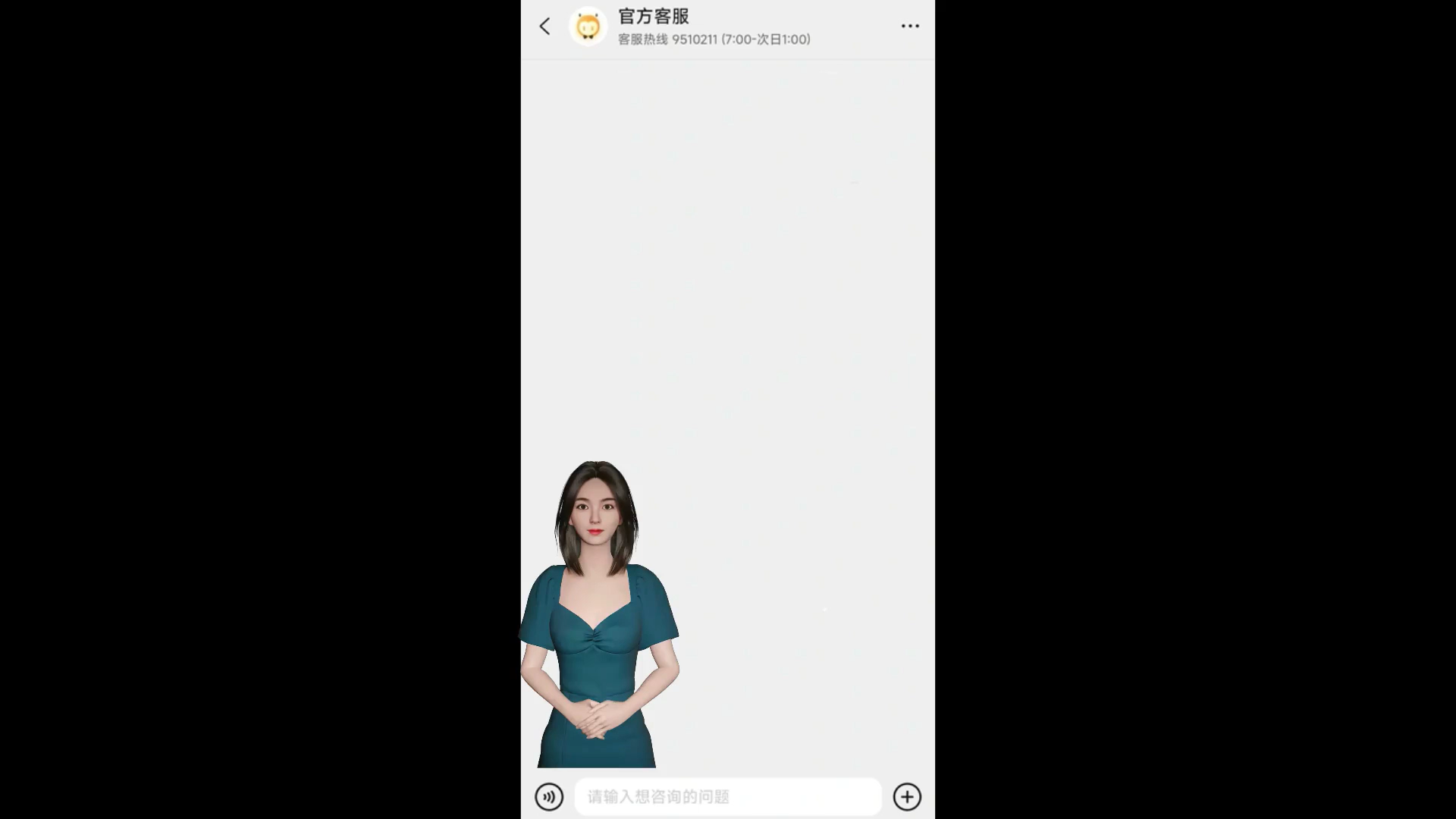 | 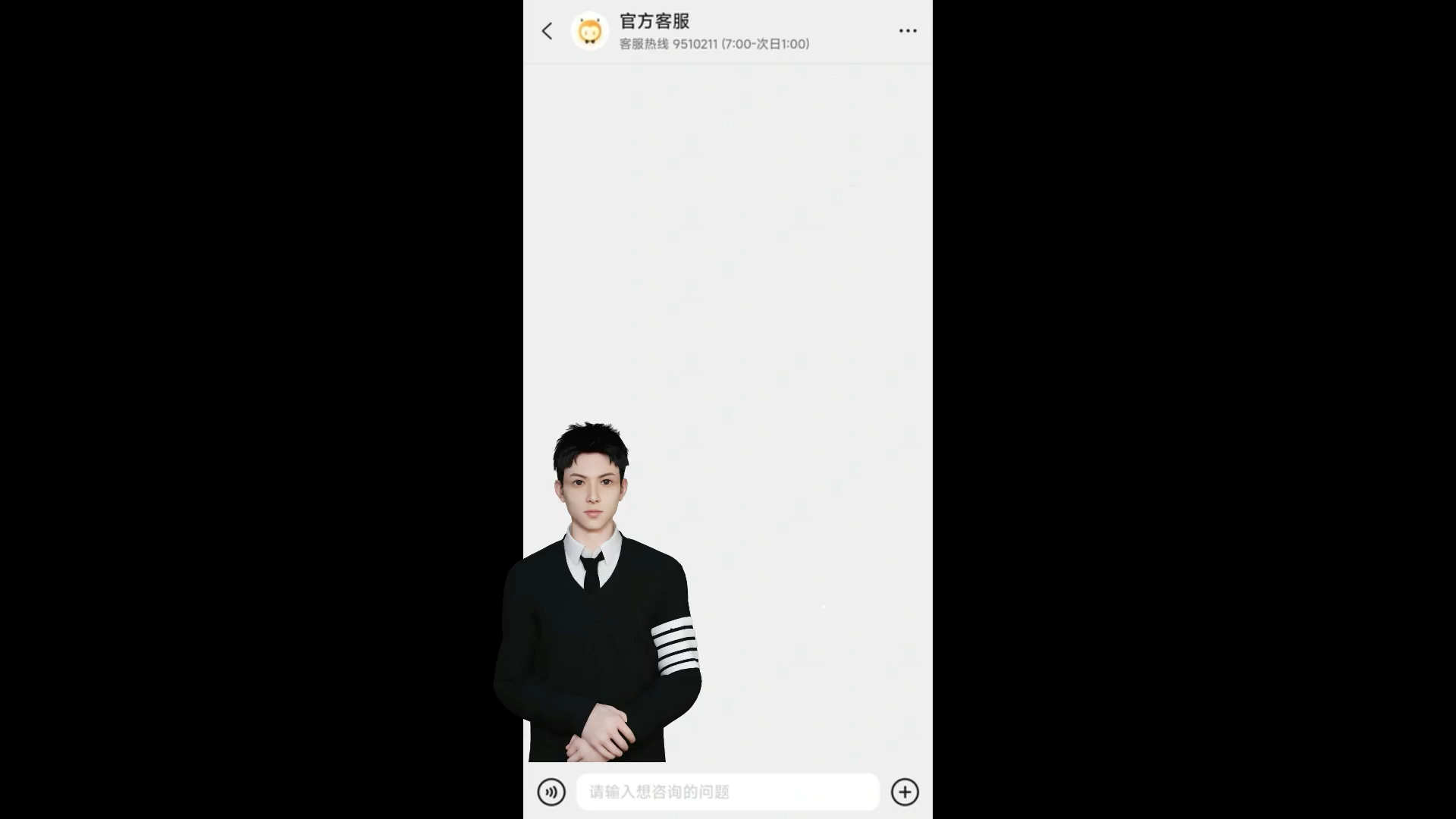 |
| For details on the virtual agent with scripted responses videos, please refer to <https://www.bilibili.com/video/BV13m421j7oT/>.  For details on the virtual agent with natural responses videos, please refer to <https://www.bilibili.com/video/BV12M4m1Q7wM/>. | For details on the virtual agent with scripted responses videos, please refer to <https://www.bilibili.com/video/BV1dsbCe2E7h/>.  For details on the virtual agent with natural responses videos, please refer to <https://www.bilibili.com/video/BV1isbCe2Ew4/>. |

# Appendix B：

Measurement Scales

| **Willingness to Use (Xie and Lei，2022; Study 1, 2, and 3)**  I intend to use this virtual agent.  I will always try to use this virtual agent.  I am willing to use this virtual agent. |
| --- |
| **Cognitive Trust (Wang et al, 2016; Study 2 and 3)**  The virtual agent is competent and effective in providing digital camera recommendations.  The virtual agent performs its role of giving recommendations very well.  Overall, the virtual agent is a capable and proficient Internet digital camera recommendation provider.  In general, the virtual agent is very knowledgeable about digital cameras.  I believe that the virtual agent would act in my best interest.  If I required help, the virtual agent would do its best to help me.  The virtual agent is interested in my well-being, not just its own.  The virtual agent is truthful in its dealings with me.  I would characterize the virtual agent as honest.  The virtual agent would keep its commitments.  The virtual agent is sincere and genuine. |
| **Affective Trust (Wang et al, 2016; Study 2 and 3)**  When I shared my tasks with the virtual agent, I felt it responded caringly.  When interacting with the virtual agent, I felt it displayed a warm and caring attitude towards me. |
| **Trait Loneliness (Pieters, 2013; Study 3)**  I feel in tune with the people around me.  I lack companionship.  There is no one I can turn to.  I do not feel alone.  I am an outgoing person.  I feel left out.  I feel isolated from others.  I can find companionship when I want to.  I am unhappy being so withdrawn.  People are around me but not with me. |

# Appendix C: The Moderated-Mediation Effect Test in Study 3

## Table C.1 The Moderated-Mediation Effect Test of State Loneliness (N = 219)

|  | Willingness to Use | | | Cognitive Trust | | | Affective Trust | | |
| --- | --- | --- | --- | --- | --- | --- | --- | --- | --- |
|  | β | SE | t | β | SE | t | β | SE | t |
| Response Type | -1.23^***^ | 0.35 | -3.509 | 0.74^*^ | 0.31 | 2.401 | 0.69 | 0.36 | 1.919 |
| State Loneliness | -0.03^**^ | 0.01 | -3.319 | -0.01 | 0.01 | -1.538 | -0.02 | 0.01 | -1.874 |
| RT×SL | 0.03^***^ | 0.01 | 3.794 | 0.01 | 0.006 | 1.816 | 0.02^**^ | 0.01 | 3.166 |
| Cognitive Trust | 0.19^*^ | 0.08 | 2.330 |  |  |  |  |  |  |
| Affective Trust | 0.45^***^ | 0.07 | 6.494 |  |  |  |  |  |  |
| R² | 0.512 | | | 0.447 | | | 0.583 | | |
| F | 44.651 | | | 58.025 | | | 100.181 | | |
| Note: * *p*<0.05 ** *p*<0.01 ****p*<0.001; RT= Response Type, SL= State Loneliness. | | | | | | | | | |

## Table C.2 The Moderated-Mediation Effect Test of Trait Loneliness (N = 219)

|  | Willingness to Use | | | Cognitive Trust | | | Affective Trust | | |
| --- | --- | --- | --- | --- | --- | --- | --- | --- | --- |
|  | β | SE | t | β | SE | t | β | SE | t |
| Response Type | 4.40^***^ | 0.60 | 7.364 | 2.01^***^ | 0.51 | 3.928 | 4.98^***^ | 0.59 | 8.504 |
| Trait Loneliness | 2.16^***^ | 0.31 | 7.011 | 0.47 | 0.29 | 1.639 | 1.74^***^ | 0.33 | 5.277 |
| RT×TL | -1.58^***^ | 0.21 | -7.722 | -0.27 | 0.19 | -1.443 | -1.16^***^ | 0.22 | -5.336 |
| Cognitive Trust | 0.22^**^ | 0.07 | 3.008 |  |  |  |  |  |  |
| Affective Trust | 0.35^***^ | 0.06 | 5.420 |  |  |  |  |  |  |
| R² | 0.593 | | | 0.445 | | | 0.595 | | |
| F | 62.151 | | | 57.534 | | | 105.049 | | |
| Note: * *p*<0.05 ** *p*<0.01 ****p*<0.001; RT= Response Type, TL=Trait Loneliness. | | | | | | | | | |
